# *Trypanosoma cruzi* Needs a Signal Provided by Reactive Oxygen Species to Infect Macrophages

**DOI:** 10.1371/journal.pntd.0004555

**Published:** 2016-04-01

**Authors:** Grazielle R. Goes, Peter S. Rocha, Aline R. S. Diniz, Pedro H. N. Aguiar, Carlos R. Machado, Leda Q. Vieira

**Affiliations:** Departamento de Bioquímica e Imunologia, Instituto de Ciências Biológicas, Universidade Federal de Minas Gerais, Belo Horizonte, Minas Gerais, Brazil; Universidade Federal do Rio de Janeiro, BRAZIL

## Abstract

**Background:**

During *Trypanosoma cruzi* infection, macrophages produce reactive oxygen species (ROS) in a process called respiratory burst. Several works have aimed to elucidate the role of ROS during *T*. *cruzi* infection and the results obtained are sometimes contradictory. *T*. *cruzi* has a highly efficiently regulated antioxidant machinery to deal with the oxidative burst, but the parasite macromolecules, particularly DNA, may still suffer oxidative damage. Guanine (G) is the most vulnerable base and its oxidation results in formation of 8-oxoG, a cellular marker of oxidative stress.

**Methodology/Principal Findings:**

In order to investigate the contribution of ROS in *T*. *cruzi* survival and infection, we utilized mice deficient in the gp91^phox^ (Phox KO) subunit of NADPH oxidase and parasites that overexpress the enzyme EcMutT (from *Escherichia coli)* or TcMTH (from *T*. *cruzi*), which is responsible for removing 8-oxo-dGTP from the nucleotide pool. The modified parasites presented enhanced replication inside murine inflammatory macrophages from C57BL/6 WT mice when compared with control parasites. Interestingly, when Phox KO macrophages were infected with these parasites, we observed a decreased number of all parasites when compared with macrophages from C57BL/6 WT. Scavengers for ROS also decreased parasite growth in WT macrophages. In addition, treatment of macrophages or parasites with hydrogen peroxide increased parasite replication in Phox KO mice and *in vivo*.

**Conclusions:**

Our results indicate a paradoxical role for ROS since modified parasites multiply better inside macrophages, but proliferation is significantly reduced when ROS is removed from the host cell. Our findings suggest that ROS can work like a signaling molecule, contributing to *T*. *cruzi* growth inside the cells.

## Author Summary

The parasite *Trypanosoma cruzi* is the causative agent of Chagas’ disease, which affects 10 million people, mainly in Latin American. Macrophages are one of the first cellular actors facing the invasion of pathogens and during *T*. *cruzi* infection, produce reactive oxygen species (ROS). To deal with oxidative stress, *T*. *cruzi* has an antioxidant machinery and, to repair DNA damage triggered by ROS, this parasite possesses enzymes of the oxidized guanine DNA repair system. The understanding of the role of ROS in the infection by *T*. *cruzi* can provide us with good insights on *T*. *cruzi* biology and virulence. While some studies suggest that ROS is related to parasite control, others have demonstrated that ROS is important for proliferation of this parasite. To investigate the contribution of ROS in *T*. *cruzi* infection, we utilized mice deficient in the production of ROS (Phox KO) and parasites that overexpress the enzymes related to DNA repair. Our results show that ROS is not only important for the battle against pathogens, but suggest that ROS can also work as a signal that contributes to the growth of this parasite.

## Introduction

Macrophages are one of the first lines of defense against intracellular pathogens [1]. During *Trypanosoma cruzi* infection, these cells are activated to produce ROS, a process called respiratory burst [2–4]. The detection of infectious agents leads to activation of the membrane bound NADPH oxidase, a multi-subunit complex that utilizes NAD(P)H as an electron donor to reduce oxygen (O_2_) to superoxide (O_2_^●−^) within the phagosome. The anionic nature of O_2_^●−^ restricts its diffusion through membranes, confining its actions to the site of formation. Superoxide radicals can spontaneously or enzymatically dismutate into hydrogen peroxide (H_2_O_2_), an oxidant with higher diffusional capacity. Metal transition ions in the presence of H_2_O_2_ can generate hydroxyl radical (^●^OH), an oxidant that, owing to its high reactivity, encloses poor selectivity against cellular targets and may not be highly toxic [5,6]. Alternatively, O_2_^●−^ may react with iNOS-derived nitric oxide (^●^NO) in a diffusion-controlled reaction, to produce peroxynitrite (ONOO^−^), a cytotoxic effector molecule against *T*. *cruzi* [4,7,8].

*T*. *cruzi* has a highly efficiently regulated antioxidant machinery to deal with the oxidative burst and adapts to the conditions imposed by their digenetic life cycle [9,10]. There are different pathways to detoxify hydroperoxides, within different substrate specificities and in different compartments such as mitochondria, glycosome, endoplasmic reticulum and cytosol [11]. In this intricate network, reducing equivalents from NADPH, produced by the pentose phosphate pathway, are delivered to a variety of detoxification enzymes. This reducing equivalents are delivered from trypanothione (T(SH)_2_) to tryparedoxin (TXN) and glutathione (GSH), which transfers them to the several peroxidases. T(SH)_2_ is maintained in its reduced state by the NADPH-dependent trypanothine reductase (TcTR) [12]. Several peroxidases have been characterized: two cysteine-dependent glutathione peroxidases, one ascorbate-dependent hemoperoxidase (TcAPX) and two tryparedoxin peroxidases [13–17]. The tryparedoxin peroxidases differ in their subcellular location: a cytosolic and a mitochondrial form (TcCPX and TcMPX, respectively) and catalyze the reduction of H_2_O_2_, small-chain organic hydroperoxides and ONOO^−^ [11,18]. In the endoplasmic reticulum, TcAPX and glutathione-dependent peroxidase II (GPX-II) metabolize H_2_O_2_ and lipid hydroperoxides respectively [16,19]. There is growing evidence that this antioxidant network may play an important role in parasite virulence and success of infection [4,11,20–22].

Despite this efficient antioxidant system, the parasite macromolecules, particularly DNA, may still suffer oxidative damage that may be deleterious if not repaired. Due to its low redox potential, guanine (G) is the most vulnerable base. The oxidation of guanine results in formation of 8-oxo-7,8-dihydroguanine (8-oxoG), a cellular marker of oxidative stress [23]. When 8-oxoG assumes *syn* configuration, it is particularly mutagenic because it functionally mimics thymine. When 8-oxoG is inserted during DNA replication, it can generate double-strand breaks, making this lesion deleterious [24,25]. To repair lesions caused by 8-oxoG, most organisms possess the oxidized guanine (GO) DNA repair system (GO system). This repair pathway is composed by the enzymes MutT, MutY and MutM in bacteria [26] and by corresponding enzymes MTH1, MUTYH and OGG1 in humans [27]. Studies on *T*. *cruzi* genome have demonstrated that this parasite has homologs to the enzymes OGG1, MUTYH [28] and MutT [29]. MutM excises the oxidized base from 8-oxoG:C base pairs and the MutY excises adenine where it has been erroneously incorporated opposite to unrepaired 8-oxoG during replication [27]. The MutT enzyme catalyzes the hydrolysis of 8-oxo-dGTP in the nucleotide pool, by substitution at the rarely attacked beta-P, to yield monophosphate nucleotide and pyrophosphate. This prevents errors in DNA replication, since the monophosphate form cannot be incorporated into nascent DNA [30,31].

Although ROS is clearly involved in control of several infections, increasing evidences point to a role of ROS as promoters of infection [32]. Several works have aimed at elucidating the role of reactive oxygen species during *T*. *cruzi* infection and the results obtained are sometimes contradictory. Although some studies have suggested that ROS produced during the respiratory burst have an important role in *T*. *cruzi* control [2,4,10,33], other authors have demonstrated that ROS is important to cellular signaling and proliferation of this parasite [11,34–36]. Studies performed with bacteria [37,38], fungus [39], viruses [40–42] and *Leishmania* [43] also associated ROS and parasite proliferation. In order to investigate the contribution of ROS in *T*. *cruzi* infection, we utilized mice deficient in the gp91^phox^ (Phox KO) subunit of NADPH oxidase [44] and parasites that overexpress the enzyme EcMutT or TcMTH and are more resistant to DNA damage caused by ROS (parasites with the *E*. *coli mutT* gene and parasites that overexpresses TcMTH gene, a *T*. *cruzi* MutT homolog) [29]. We found that modified parasites multiply better inside macrophages than wild type, but their proliferation is significantly reduced when ROS production is inhibited in host cell. Our results suggest that low concentration of ROS may work like a signaling molecule, contributing to growth of *T*. *cruzi* inside the cells *in vitro* and increasing the levels of parasitemia *in vivo*.

## Materials and Methods

### Ethics statement

This study was conducted in strict accordance with the recommendations in Guide for the Care and Use of Laboratory Animals of the Brazilian National Council of Animal Experimentation (http://www.cobea.org.br/) and the Federal Law 11.794 (October 8, 2008). All animals were handled in strict accordance with good animal practice as defined by the Internal Ethics Committee in Animal Experimentation (CETEA) of the Universidade Federal de Minas Gerais (UFMG), Belo Horizonte, Minas Gerais, Brazil. The protocol number 214/11 was approved by CETEA.

### Parasites

*T*. *cruzi* epimastigotes (CLBrener, wild-type) were cultured at 28°C in BHI (brain heart infusion) medium. Parasites overexpressing EcMutT, TcMTH and parasites transfected with the empty vector pROCK (TcROCK) were obtained as described previously [29,45]. Transformed cells were cultured in BHI medium containing 250 *μ*g·ml^−1^ of hygromycin (Sigma Aldrich, St. Louis, MO, USA). *T*. *cruzi* trypomastigotes were obtained from the supernatant of infected mono layers of LLC-MK_2_ cell cultures (grown in 2% FBS, 1% penicillin-streptomycin and 2 mM glutamine supplemented DMEM (Dulbecco´s Modified Eagle´s Medium, Sigma Aldrich) and purified by incubation of the pellet for 2 hours at 37°C, followed by collection of motile infective trypomastigotes in the supernatant. This project was approved by National Technical Biosafety Commission (CTNBio) under the process number: 01200.003883/97-02.

### Animals

Four- to 8-week-old male and female C57BL/6 mice were obtained from CEBIO (Instituto de Ciências Biológicas, Universidade Federal de Minas Gerais.Belo Horizonte, MG, Brazil). Phox KO [44] and IFN-γ KO [46] mice were purchased from The Jackson Laboratory (Glenville, NJ, USA). Mice were kept in conventional conditions with barriers, controlled light cycle and controlled temperature. Animals were fed a commercial diet for rodents (Labina, Purina, SP, Brazil) *ad libitum*.

### *In vitro* assays for parasite burden

The macrophages used in this study were isolated from the peritoneal cavity of mice 4 days after injection of 2 mL of 3% thioglycollate medium (BD, Le Pont de Claix, France) into the peritoneal cavity. After this time, mice were euthanized and the peritoneum cells were harvested by repeated cycles of aspiration and re-injection with 10 ml of cold PBS in 10ml syringe with a 24G needle. More than 80% of the cells harvested were macrophages. The cells were centrifuged at 4°C, 1,500 g for 10 minutes and re-suspended in DMEM supplemented with 10% fetal bovine serum (FBS) (Cultilab, Campinas, SP, Brazil), 1% penicillin-streptomycin and 2mM glutamine. Macrophages were counted in a hemocytometer prior to seeding 5x10^5^ or 1x10^6^ cells into each well of a 24-well or 72-well plate respectively and incubated at 37°C, 5% CO_2_ for 2 hours. The parasites were purified, counted and diluted in DMEM medium, and infection was performed for 2 hours, at a five-parasite-to-one-macrophage ratio. Immediately after macrophage infection, the cells were washed four times with phosphate-buffer saline (PBS, pH 7.3) to remove extracellular parasites. The cells were fixed or reincubated with medium for 48 and 72 hours before fixation with methanol. Coverslips with attached macrophages were stained with Panótico (Laborclin, Pinhais, PR, Brazil) and a minimum of 300 macrophages per coverslip were counted. The results were expressed as an infection index ([percentage of infected macrophages x number of amastigotes]/total number of macrophage). Cells from 96 well plates were used to count released parasites in the supernatant (3–7 days after infection). The following drugs were used in these assays: apocynin (APO) (300μM; Sigma-Aldrich); N-acetyl-cysteine (NAC) (1mM; Sigma-Aldrich); H_2_O_2_ (100 μM); superoxide dismutase–polyethylene glycol (SOD) (25 U/well, Sigma-Aldrich) and catalase–polyethylene glycol (CAT) (40 U/well, Sigma-Aldrich). Drugs were added to the cells 30 minutes (H_2_O_2_) or 2 hours (apocynin, catalase, NAC, SOD-PEG) before and immediately after infection. Parasites were treated with 100μM H_2_O_2_ for 30 minutes before the infection.

### ROS detection

Luminometry assays were performed to evaluate the production of ROS by macrophages. The cells, obtained as described before, were centrifuged at 4°C, 1,500 *g* for 10 minutes, and resuspended in complete RPMI without phenol red. Macrophages (1 x 10^6^ cells/well) were plated in 96 well opaque plates (NUNC, Rochester, NY, USA) and pre-incubated with 300μM of APO, 1mM of NAC, 25u of SOD or 40u of CAT for 2 hours. After this time, 0.05 mM luminol (5-Amino-2,3-dihydro-1,4-phthalazinedione; Sigma-Aldrich) and *T*. *cruzi* trypomastigotes (Y strain) in the proportion of 10 parasites to 1 macrophage were added in each well. Measurements were taken for 120 minutes with two-minute interval between measurements. Production of ROS was assayed by the light intensity generated by the reaction between ROS and luminol and expressed as relative light units.

### ^●^NO production by macrophages

Macrophage iNOS was induced by pre-incubating the cells with 100 units/mL of IFN-γ (BD, San Diego, CA, USA) and 10μg/mL of LPS (Invivogen, San Diego, CA, USA) for 2 hours. Then, control and IFN-γ/LPS-activated macrophages were infected with *T*. *cruzi* trypomastigotes (5 parasites: 1 host cell) for 2 hours and washed with phosphate-buffer saline (PBS, pH 7.3) to remove extracellular parasites. Following incubation (for 48 h), supernatants were collected and the concentration of nitrite was determined spectrophotometrically (Microplate Spectrophotometer System, model SPECTRAmax 340, Molecular Devices, Sunnyvale, CA, USA) at 540nm using the Griess method with NaNO_2_ as the standard [47].

### DHR (dihydrorhodamine) oxidation

*T*. *cruzi* epimastigotes (TcWT, EcMutT and TcMTH) were treated with 200μM of H_2_O_2_ for 30 minutes. After incubation, cells were centrifuged at 800 *g* for 10 min at 25°C and washed twice in DPBS (Dulbecco’s PBS, pH 7.3; Sigma-Aldrich). Parasites (1 × 10^9^ cells/mL) were incubated for 30 min at 28°C in DPBS containing 50 *μ*M DHR (Molecular Probes, Life Technologies, Eugene, OR, USA). After incubation, cells were centrifuged at 800 *g* for 10 min at 25°C and washed twice in DPBS in order to eliminate non-incorporated DHR. Detection of intracellular Rhodamine 123 (RH 123), the oxidation product of DHR, was performed after exposure to the 0.1mM peroxynitrite donor 3-morpholinosydnonimine hydrochloride (SIN-1, Sigma-Aldrich). The detection of intracellular RH 123 was performed using a FACS-Calibur flow cytometer (Becton-Dickinson, Rutherford, NJ, USA).

### Detection of antioxidants enzymes in parasites

Epimastigotes (3x10^8^ cells) were replicated 3 days consecutively to maintain parasites in the logarithmic phase of growth and after this process were incubated with 50μM H_2_O_2_ for 30 minutes, washed twice and prepared for determination of antioxidant enzyme contents. Parasites (3x10^8^ cells) were centrifuged at 800 *g* for 10 min at 25°C, washed three times in DPBS pH 7.3, re-suspended in 250 μL lysis buffer (10 mM Tris–HCl, 1 mM EDTA and 0.5% (v/v) Triton X-100) and incubated on ice for 15 min. Cell extracts were clarified (13,000 *g* for 30 min at 4°C) and supernatants supplemented with loading buffer (30 mM Tris–HCl, pH 6.6, 1% (w/v) SDS and 5% (v/v) glycerol) were stored at -80°C until use. Protein extracts (50 μg), were resolved by 15% SDS–PAGE and then blotted into nitrocellulose membranes (Hybond-C extra, GE Healthcare Life Sciences, USA). After transfer, proteins were stained with Ponceau-S solution (Aplichem, Daermstadt, Germany) and blocked using 3% dry milk in PBS for 1 h at 25°C. Membranes were then probed with anti-TcCPX (1:2000) diluted in PBS 0.1% (v/v) Tween 20 for 1 h at 25°C following 1 h incubation with anti-rabbit IRE-800 (LI-COR, Lincoln, NE, USA) diluted 1:10.000 in PBS 0.1% (v/v) Tween 20. Membranes were imaged with the LI-COR Odyssey Infrared Imaging System. Protein content relative to total protein loaded (Ponceau-S staining [48]) in the different extracts analyzed was determined by densitometric techniques using ImageJ (National Institute of Health, Bethesda, MD, USA). Results are expressed as relative enzyme content respect to total protein content [20]. To determinate the quantity of TcMPX and TcSODB, the parasites were fixed in paraformaldehyde (4% v/v in PBS) and incubated with anti-TcMPX, anti-SODB, and anti-cruzipain (1:2000) for 1 hour at 37°C. The parasites were washed and incubated for 1 h with Alexa Fluor 488 goat anti-rabbit IgG (Life Technologies, Eugene, OR, USA) diluted 1:10,000, washed again and analyzed by flow cytometer (FACS-Calibur).

### 8-oxoG assay

Streptavidin has previously been shown to bind with high specificity to 8-oxoG [49,50] and was therefore used for the 8-oxoG measurements. Epimastigotes and amastigotes parasites were treated with H_2_O_2_ for 30 minutes, fixed in paraformaldehyde (2% v/v in PBS) at 25°C for 15 min and thereafter incubated for 15 min in PBS with 0.1% Triton X-100 v/v. Cells were then incubated with Alexa488-conjugated streptavidin (Invitrogen) (1:100) in PBS for 1 h at 37°C and evaluated by flow cytometer (FACS-Calibur).

### *In vivo* infection experiments

*T*. *cruzi* trypomastigotes were maintained by blood passage in IFN-γ KO (TcWT and TcMTH strain) or Swiss (Y strain) mice every 7 or 9 days respectively. Trypomastigotes were obtained from heparinized blood, counted and used to infection. In some experiments, blood parasites (Y strain) were treated with 100μM H_2_O_2_ for 30 minutes before the infection. Experimental infection was performed in C57BL/6 WT and Phox KO mice by intraperitoneal injection of 10^6^ TcWT, TcMTH or Y strain blood trypomastigotes. Parasitemia was assessed by counting trypomastigotes in 5 μL of tail vein blood, every day from the 3^rd^ day post-infection until the time at which the parasites became undetectable. The number of parasites per mL was calculated as previously described [51]. Mortality of infected mice was monitored daily.

### Statistical analysis

Statistical analysis in this work was performed using the GraphPad Prism 5.0 program (GraphPad Software Inc., CA, USA). Data are presented as the mean ± standard deviation (SD), and all experiments were repeated at least three times. Data were analyzed for significant differences using ANOVA, and differences between groups were assessed with Bonferroni post-test. The level of significance was set at p < 0.05.

## Results

### *T*. *cruzi* triggers ROS production by macrophages

**Fig 1:**
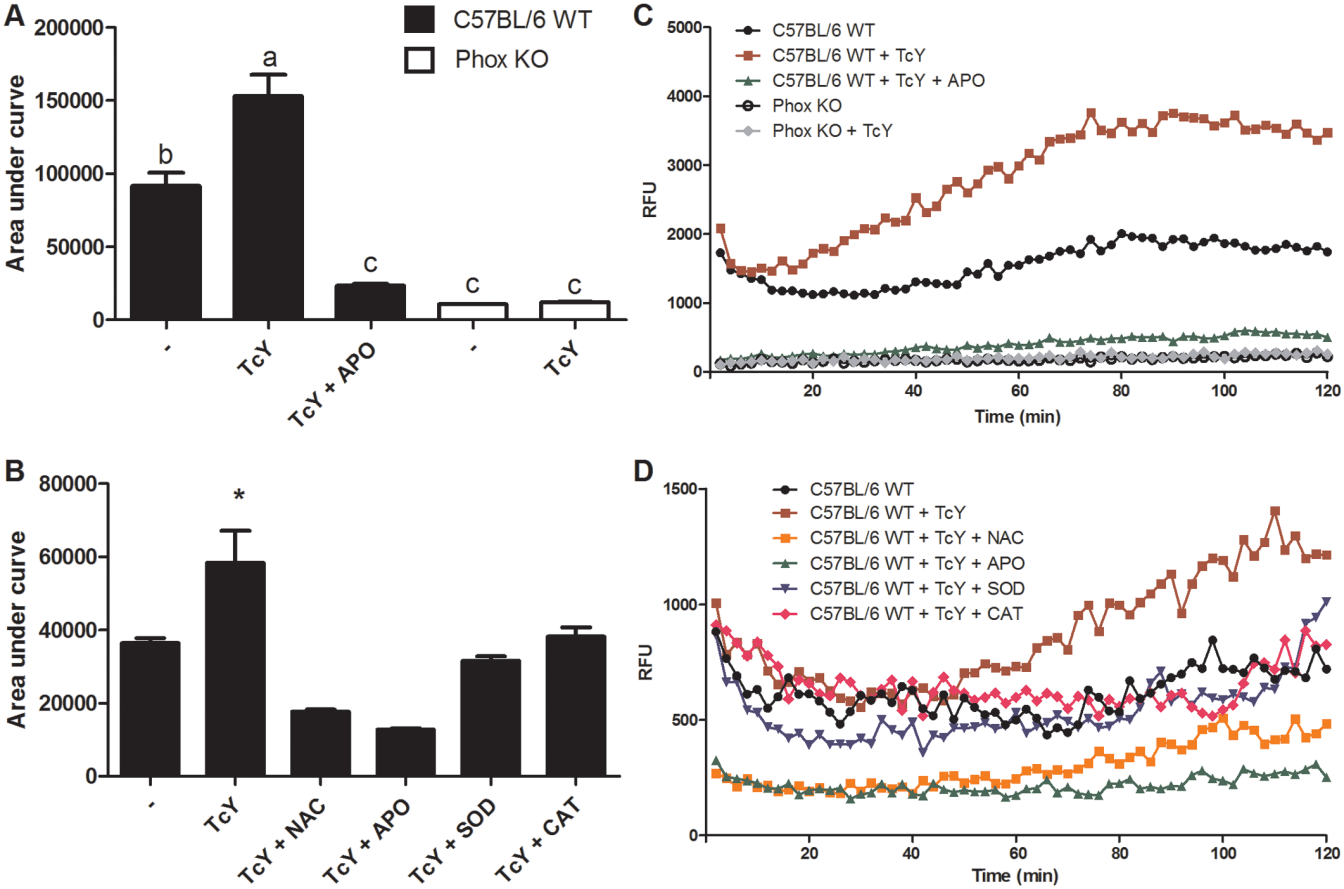
Production of reactive oxygen species by macrophages stimulated with Y strain of *T*. *cruzi*. Thioglycolate-elicited macrophages were harvested from the peritoneal cavity of C57BL/6 WT and Phox KO mice 4 days after stimulation. Reactive oxygen species production by macrophages was detected by luminol. Macrophages previously incubated with apocynin (APO), catalase (CAT), superoxide dismutase (SOD) and N-acetyl-cysteine (NAC) for 2 hours were incubated with 0.5mM of luminol in culture medium and exposed to *T*. *cruzi* trypomastigotes. Chemiluminescence was continuously measured immediately after *T*. *cruzi* addition to the macrophage monolayer, and the area under the obtained curves was calculated. (A, C) Graphs showing area under curve, data represent mean of triplicates ± S.D. of total counts in 120 min. (B, D) Graphs showing chemiluminiscence rates, data represent means of triplicate counts in 120 min, standard deviations were omitted for clarity. The graphs are representative of five independent experiments performed in triplicate (cells were pooled from three mice for each replicate). * refers to significant differences from the infected non-treated macrophages. Bars marked by different letters are statistically different (p<0.05, one-way ANOVA test with Bonferroni post-test).

Internalization of *T*. *cruzi* trypomastigotes by macrophages triggers the assembly of the NADPH oxidase complex to yield O_2_^●−^ [4]. To establish that infection promotes respiratory burst in our conditions, we performed chemiluminescence experiments using luminol which can serve as a probe for O_2_^●−^ and ONOO^−^ [4]. Infection-increased chemiluminescence triggered by parasites was almost twice that observed with non-infected cells (Fig 1). Luminol chemiluminescence increase was not detected when we performed these experiments in Phox KO macrophages, due to the lack of phagocyte NADPH oxidase (phox) activation and thus O_2_^●−^ production (Fig 1A and 1C). As expected, pretreatment of macrophages with the phox inhibitor apocynin, a compound that prevents p47phox subunit translocation and therefore assembly of the enzyme complex, prevented the increase in ROS induced by infection, and brought chemiluminescence values down, similarly to Phox KO macrophages (Fig 1A and 1B). Addition of the antioxidants NAC, SOD and CAT also reduced chemiluminescence intensity (Fig 1B and 1D). Once determined in our experimental setup that *T*. *cruzi* could stimulate ROS production by macrophages, we proceeded to investigate if oxidative stress would affect the course of infection.

### Parasites with enhanced 8-oxo-dGTP pyrophosphohydrolase activity presented improved growth in macrophage cultures

**Fig 2:**
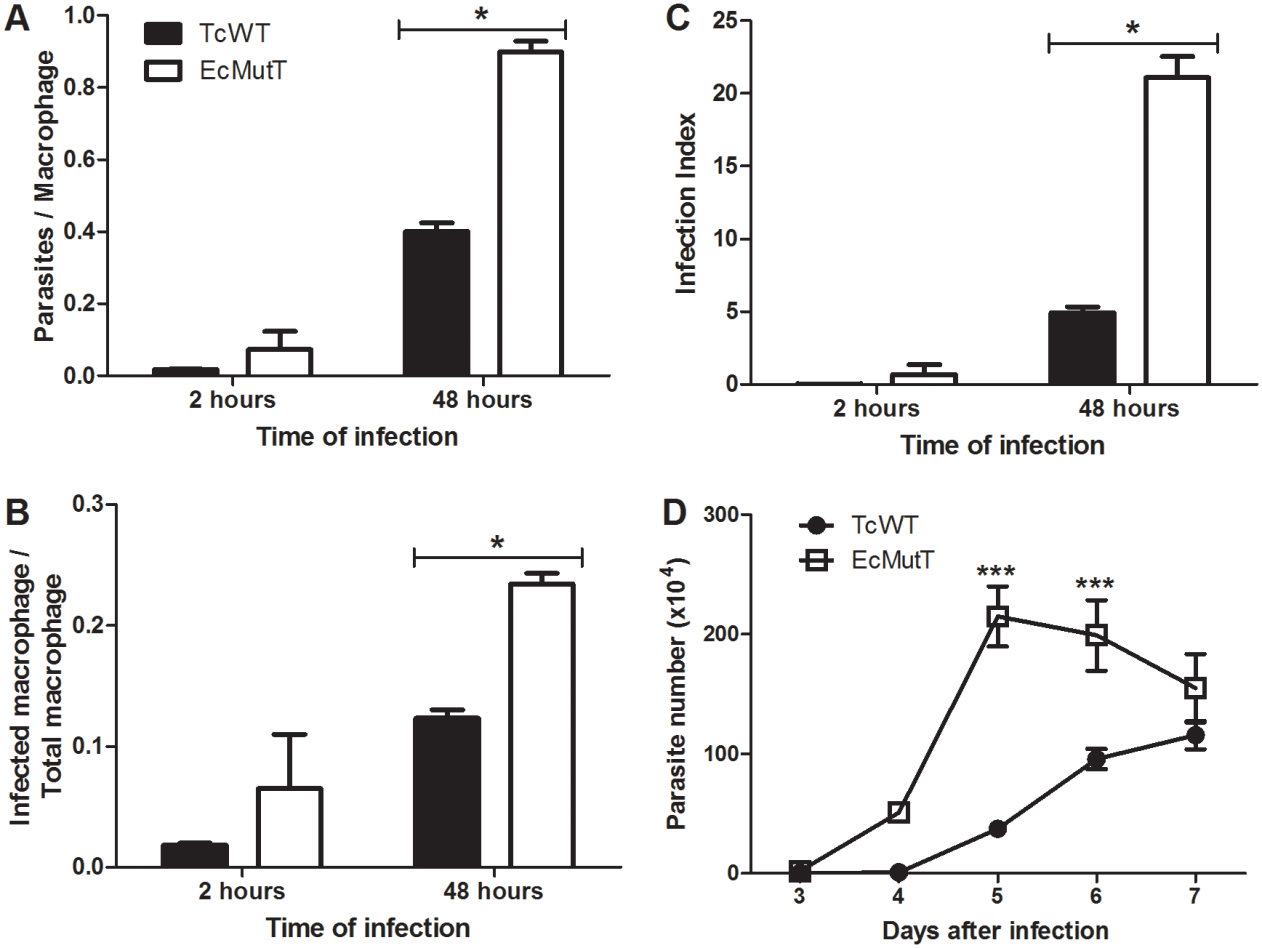
Growth of parasites with enhanced 8-oxod-GTP pyrophosphohydrolase activity in macrophages. Inflammatory macrophages obtained from peritoneal cavity of C57BL/6 WT mice were infected with wild type and modified parasites. The cells were washed to remove extracellular parasites and either fixed or re-incubated with medium for different times. **(A)** Number of parasites per macrophage. **(B)** Number of infected macrophages per total macrophages. **(C)** Infection index for each parasite population. **(D)** Number of parasites released into the macrophage culture supernatant between the third and seventh day after infection. Data shown are from one representative of three independent experiments performed in triplicate (cells were pooled from three mice for each replicate). All data are presented as the mean ± standard deviation of triplicates. * indicates significant differences between marked bars or points, p<0.05, two-way ANOVA test with Bonferroni post-test. A replicate of this experiment is presented in S1 Fig.

To investigate the importance of oxidative stress on the success of infection of macrophages with *T*. *cruzi*, we used parasites that over-express EcMutT enzyme and are more resistant to DNA damage by the oxidation of guanine [29]. To investigate the influence of over-expression of MutT/MTH on *T*. *cruzi* invasion process in host cells, macrophages were exposed to parasites for two hours, washed to eliminate extracellular parasites, and fixed. EcMutT heterologous expression does not affect the invasion process (Fig 2A, 2B and 2C). The number of internalized trypomastigotes (Fig 2A) and the number of infected macrophages (Fig 2B) was similar between the two populations of parasites. However, after 48 hours, the number of infected macrophages (Fig 2B) and the number of amastigotes per macrophage (Fig 2A) was elevated for EcMutT parasites in comparison with TcWT parasites. To better express the obtained data the infection index was determined, considering simultaneously the number of infected macrophages and the number of amastigotes in relation to total macrophages. The infection index shows that EcMutT presented increased replication inside murine inflammatory macrophages when compared with wild-type parasites (Fig 2C). This enhanced replication of EcMutT parasites inside macrophages was corroborated by counting the number of trypomastigotes released at the supernatant of infected cells (Fig 2D). Hence, removal of 8-oxo-dGTP from the nucleotide pool increased the success of *T*. *cruzi* inside murine macrophages. A replicate of this experiment is presented in S1 Fig.

### TcMTH and EcMutT parasites effectively decreased peroxynitrite-dependent intracellular DHR oxidation and show increase in antioxidant enzyme expression

**Fig 3:**
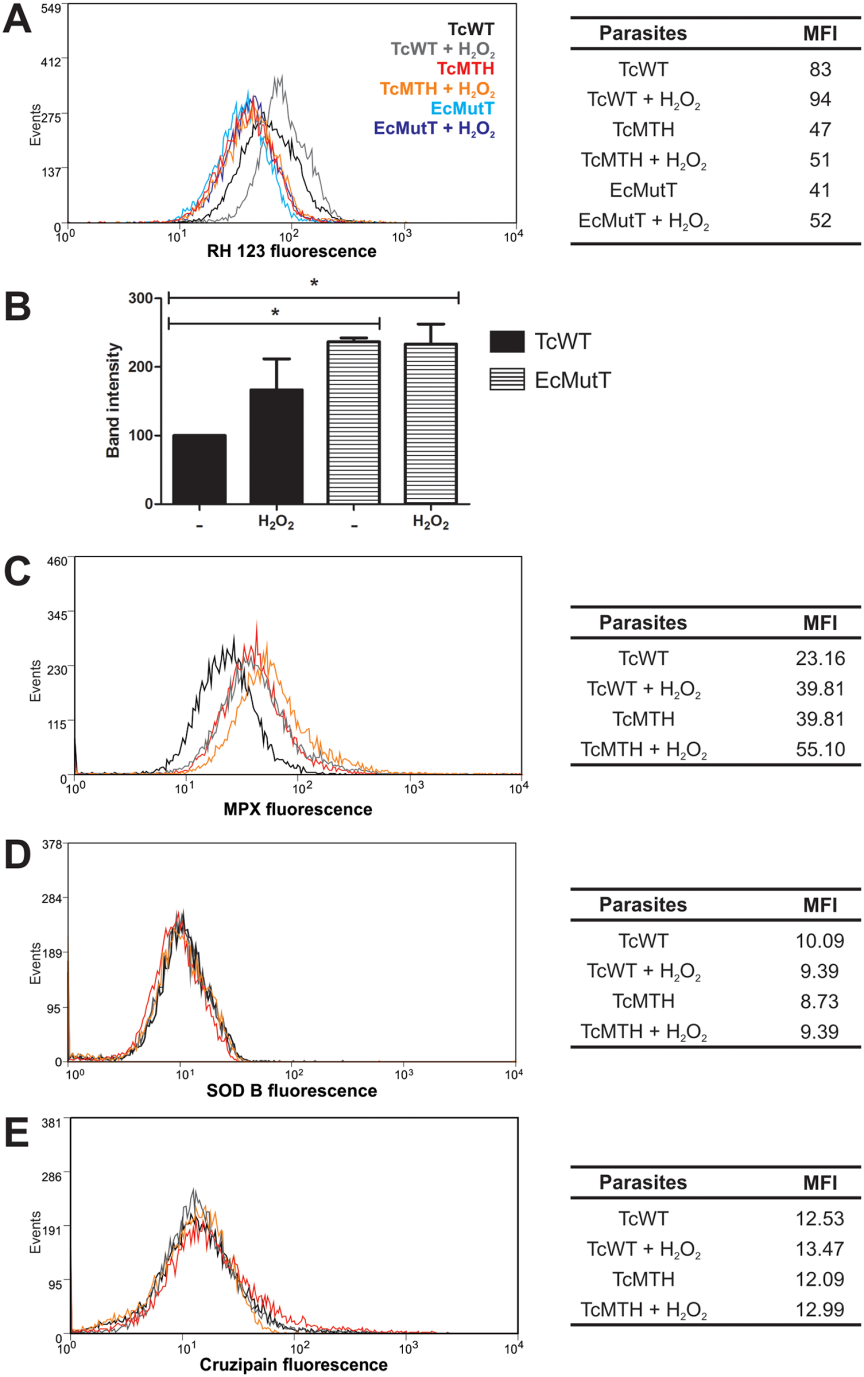
TcMTH and EcMutT parasites effectively decreased DHR oxidation and show increase in antioxidant enzyme expression. *T*. *cruzi* epimastigotes (TcWT, EcMutT and TcMTH) were treated with 200μM H_2_O_2_ for 30 minutes. **(A)** Parasites were pre-loaded with DHR (50 μM), exposed to the peroxynitrite donor 3-morpholinosydnonimine hydrochloride (SIN-1, 0.1mM) and intracellular RH 123 was measured by flow cytometry. Histogram overlays and MFI values plotted in the table are representative one of three independent experiments with similar results. **(B)** Cytosolic tryparedoxin peroxidase in parasite extracts was detected by Western Blot using an anti-TcCPX specific antibody. Results are expressed as the density of bands in the Western Blot, pooled results from three experiments performed. A representative Western Blot is in S2 Fig. **(C)** Mitochondrial tryparedoxin peroxidase, **(D)** superoxide dismutase B and **(E)** cruzipain were detected by anti-TcMPX, anti-SODB, and anti-cruzipain specific antibodies, respectively, by flow cytometry. Histogram overlays and MFI values plotted in the table are representative one of three independent experiments with similar results.

EcMutT parasites express *E*. *coli* MutT enzyme, whereas TcMTH parasites overexpress a *T*. *cruzi* MutT homolog [29]. Both parasites multiply better in macrophages than wild-type (TcWT) and the wild-type parasite transfected with the empty vector pROCK (TcROCK) (Fig 2 and [29]). To determine if EcMutT and TcMTH are resistant to oxidative stress and what would be the mechanism for this resistance, we evaluated DHR oxidation by flow cytometry in parasites exposed to peroxynitrite donor, SIN-1. Oxidation of the DHR loaded into epimastigotes into fluorescent rhodamine 123 indicates that SIN-1 reaches the parasite cytosol (Fig 3A). The highest intracellular DHR oxidation yield was obtained when TcWT epimastigotes were pre-incubated with H_2_O_2_. DHR oxidation was not increased in TcMTH and EcMutT pre-incubated with hydrogen peroxide and further challenged with peroxynitrite (Fig 3A). We observed that pre-conditioning with H_2_O_2_ did not promote increase in expression of cytosolic tryparedoxin peroxidase (Fig 3B and S2 Fig gel), but increases expression of mitochondrial tryparedoxin peroxidase (Fig 3C and [29]) in both TcWT and TcMTH. Interestingly, EcMutT over-expressed cytosolic tryparedoxin peroxidase. Superoxide dismutase B (Fig 3D) was similar among parasites and after pre-treatment with H_2_O_2_. Cruzipain expression was used as control (Fig 3E). Treatment with H_2_O_2_ did not alter parasite viability (S3 Fig).

### MTH prevents 8-oxo-dGTP incorporation into DNA

**Fig 4:**
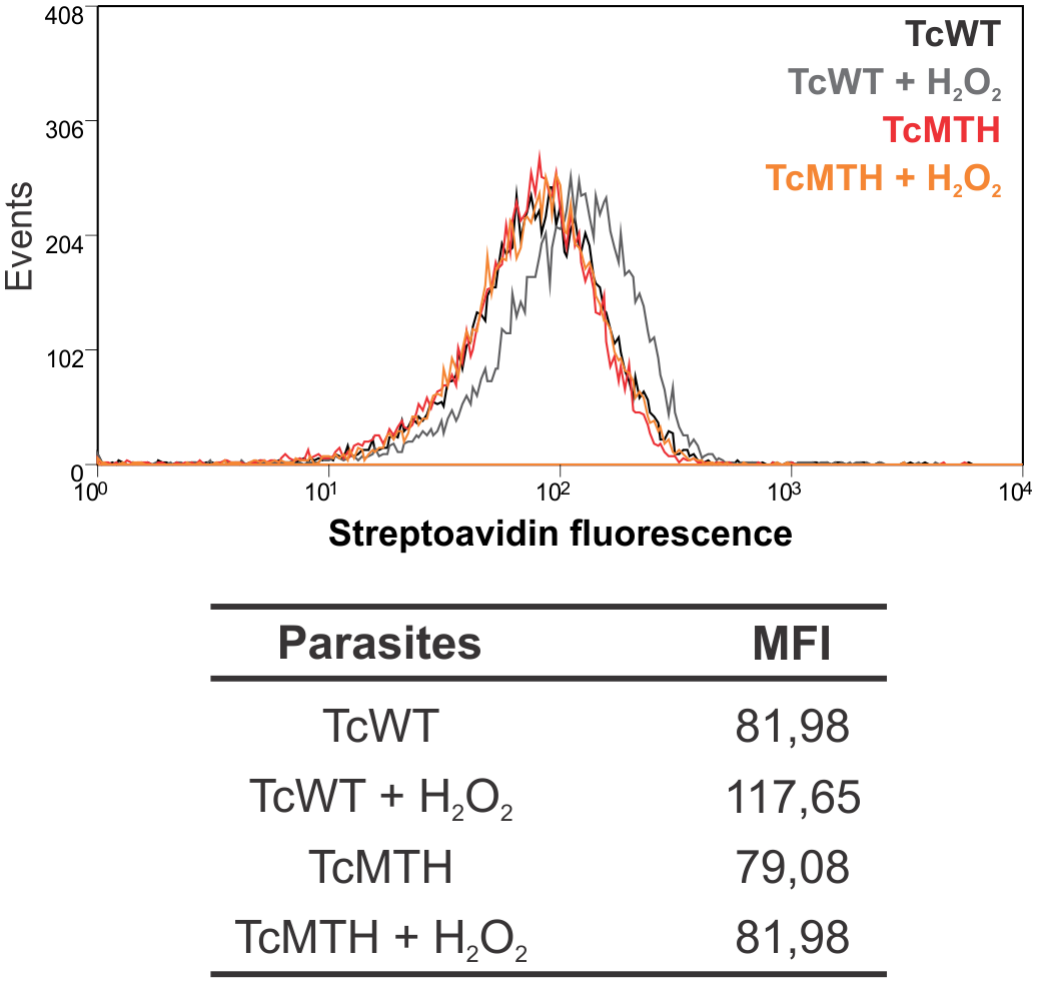
The enzyme MTH prevents 8-oxodGTP incorporation. Epimastigotes were treated with 200μM H_2_O_2_ for 30 minutes, fixed and 8-oxoG incorporation in DNA was evaluated with streptavidin-Alexa Fluor 488 by flow cytometer. Histogram overlays and MFI values plotted in the table are from one representative experiment three independent experiments with similar results.

We had already demonstrated that MutT/MTH-expressing cells contained fewer nuclear DNA lesions [29]. We now evaluated the accumulation of 8-oxoG in DNA after parasite exposure to H_2_O_2_. The modified base was detected using streptavidin conjugated with Alexa-488 by flow cytometer. We observed an increase of 8-oxoG in DNA after H_2_O_2_ treatment in TcWT epimastigotes. On the other hand, TcMTH epimastigotes did not show increased 8-oxoG in DNA after H_2_O_2_ treatment, demonstrating the functional activity of the MTH enzyme in the *T*. *cruzi* over-expressers (Fig 4).

### Phox KO macrophages display reduced parasitism

**Fig 5:**
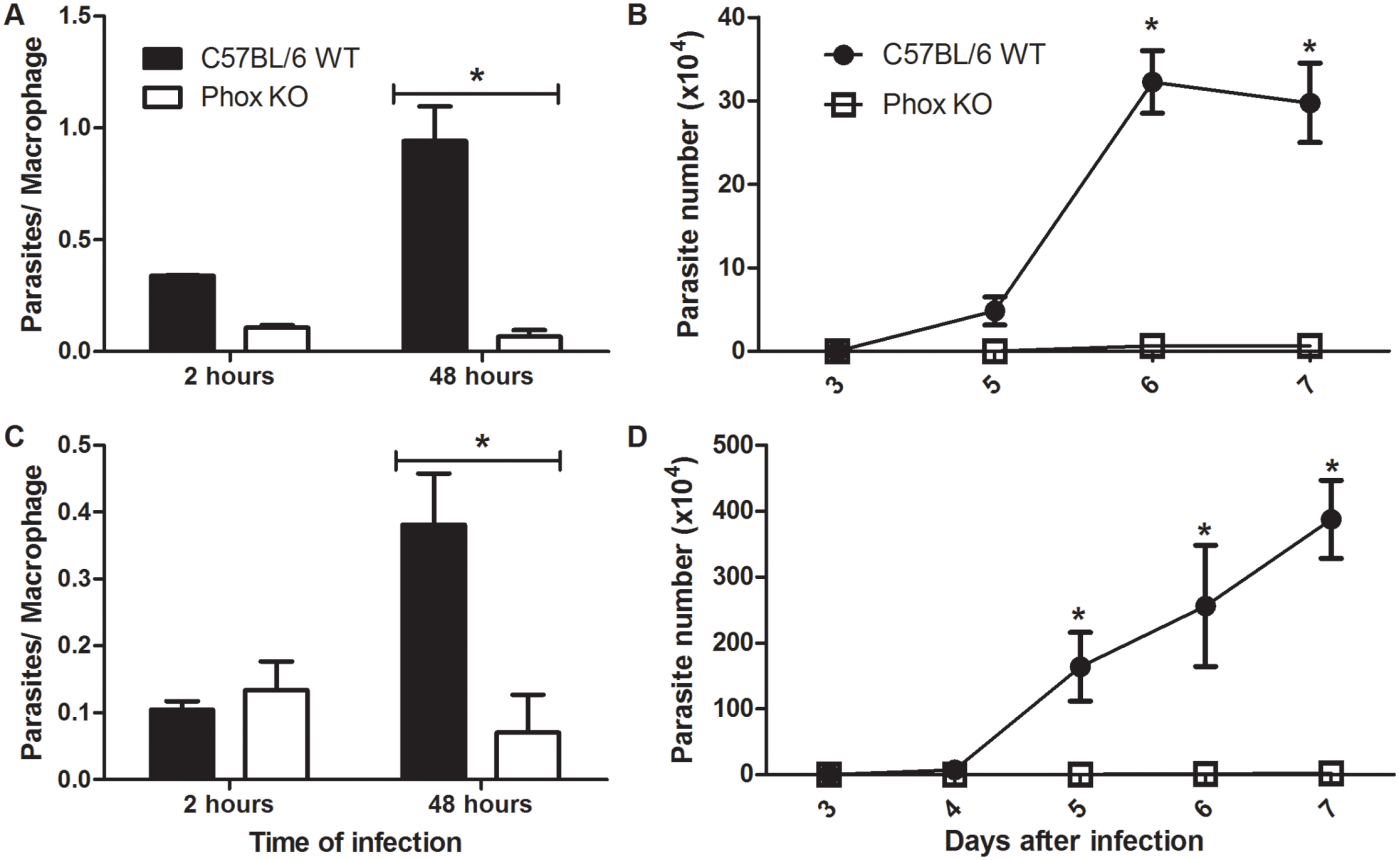
Phox KO macrophages present reduced parasitism. Inflammatory macrophages obtained from peritoneal cavity of C57BL/6 WT and Phox KO mice were subjected to infection with Y **(A, B)** and CL Brenner **(C, D)** strains of *T*. *cruzi*. Cells were washed to remove extracellular parasites and either fixed or re-incubated with medium for different times. The slides were stained and counted to determine the number of parasites per macrophage. A minimum of 200 macrophages were counted per group in triplicate. **(A, C)**. The number of parasites released in the supernatant of cells between the third and seventh day after infection was quantified **(B, D)**. Data shown are representative one of three independent experiments performed in triplicate. All data are presents as the means ± standard deviation. * indicates significant differences between marked bars or points, p<0.05, two-way ANOVA test with Bonferroni post-test.

**Fig 6:**
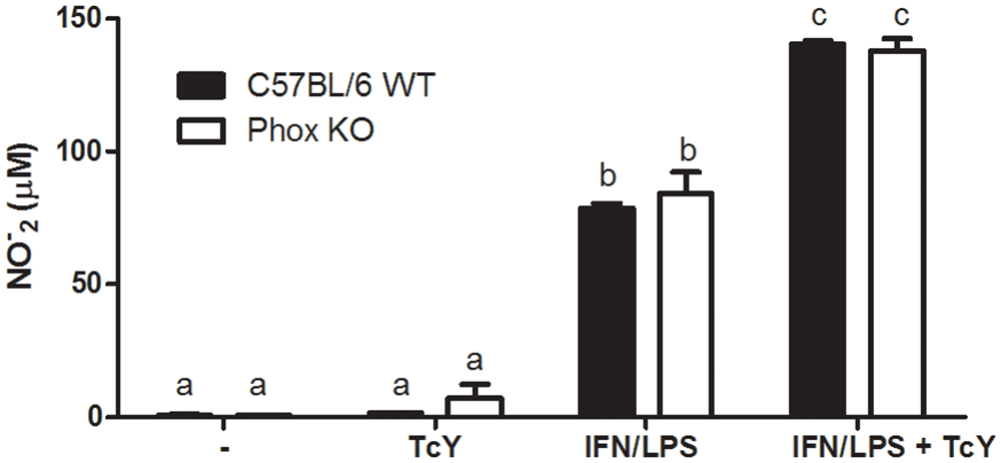
NO production by Phox KO and C57BL/6 WT macrophages. Inflammatory macrophages obtained from peritoneal cavity of C57BL/6 WT and Phox KO mice were stimulated with IFN-γ/LPS and infected with *T*. *cruzi*. After 48 hours, NO production was quantified by the Griess reaction.

Our results so far indicate that the over-expression of genes related to repair of oxidative damage favors the growth of parasites inside macrophages. To understand better the role of ROS in *T*. *cruzi* infection, we infected macrophages from Phox KO mice. Macrophages from these mice produced less ROS than cells from C57BL/6 WT mice upon infection (Fig 1A and 1C). Phox KO macrophages showed reduced parasitism, when infected with Y (Fig 5A and 5B) and CL Brenner (Fig 5C and 5D) strains of *T*. *cruzi*, as compared to C57BL/6 WT macrophages. After 48 hours of infection with Y strain, the number of parasites was increased in C57BL/6 WT macrophages, but was reduced in Phox KO macrophages (Fig 5A). In addition, the number of trypomastigotes released in the supernatant of C57BL/6 WT infected macrophages was greater than in Phox KO macrophages after infection with the Y strain (Fig 5B). The same result was obtained with the CL Brenner strain of *T*. *cruzi* (Fig 5C and 5D). We did not observe significant differences in ^●^NO production between Phox KO and C57BL/6 WT macrophages. In both cells, infection with *T*. *cruzi* did not induce the production of ^●^NO, which was similar to basal levels obtained by non-infected cells. The treatment with IFN-γ/LPS induced significant amounts of ^●^NO both in Phox KO and C57BL/6 WT macrophages. ^●^NO production was not different between Phox KO and C57BL/6 WT submitted to the same treatment. Additionally, ^●^NO production by previously stimulated cells was increased by *T*. *cruzi* infection (Fig 6).

### The inhibition of ROS production by macrophages also impairs *T*. *cruzi* proliferation

**Fig 7:**
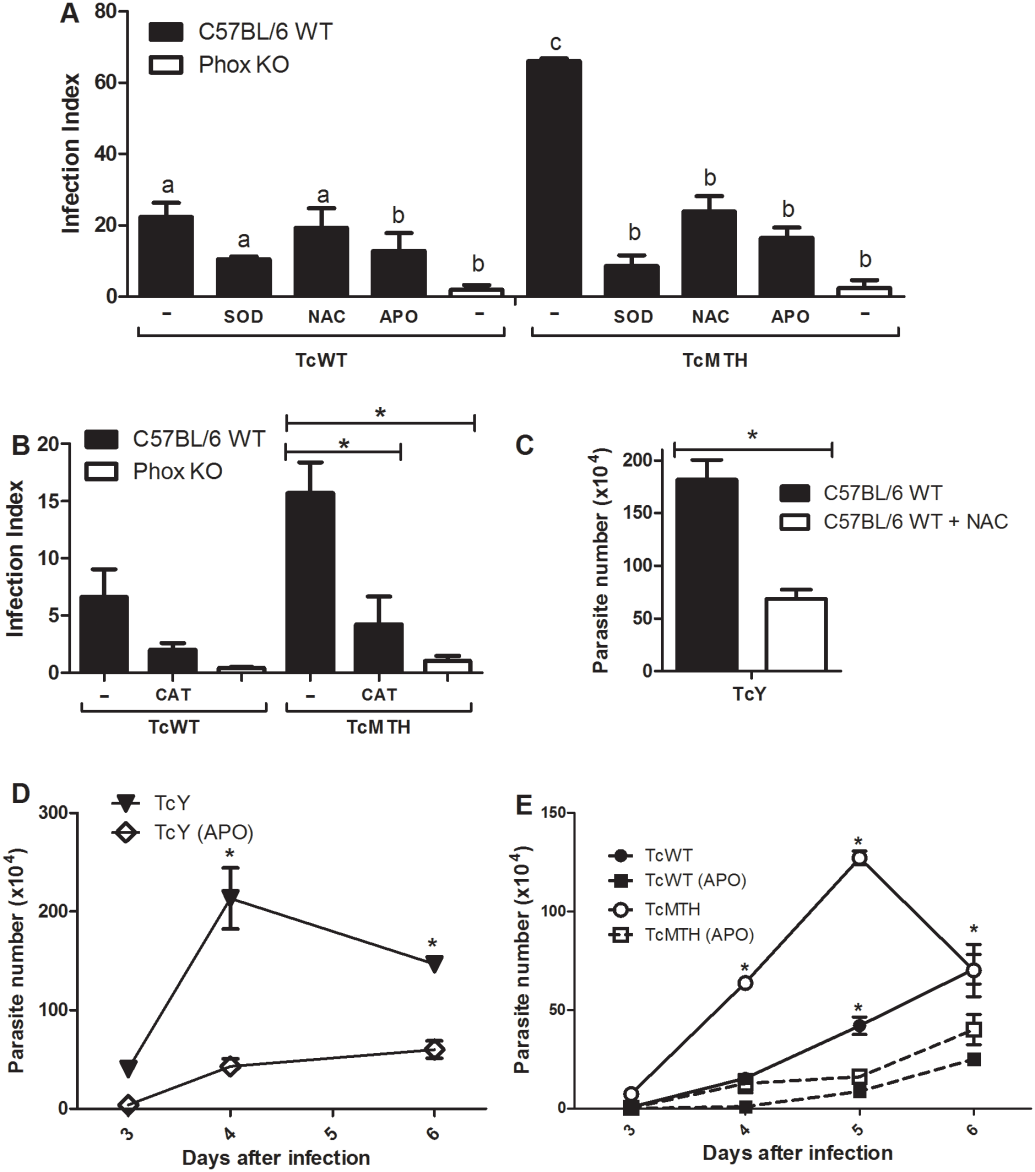
Inhibition of ROS production in macrophages impairs *T*. *cruzi* proliferation. Inflammatory macrophages from C57BL/6 WT and Phox KO mice were incubated with 300μM of apocynin (APO), 1mM of N-acetyl-cysteine (NAC), 25U of superoxide dismutase-polyethylene glycol (SOD) and catalase-polyethylene glycol (CAT) 2 hours before the infection with different parasites. Parasites were added for another 2 hours, the cells were washed to remove extracellular parasites, and ROS inhibitors were added back to the cultures. **(A,B)** Slides were stained and counted to determine the infection index 72 hours after infection. A minimum of 200 macrophages were counted per group in triplicates. The number of Y strain parasites (**C, D**) and TcWT, TcMTH **(E)** in supernatants was quantified four days after infection **(C)** or between the third and sixth day after infection **(D, E)**. Data shown are representative one of three independent experiments performed in triplicates. All data are presented as the means ± standard deviation. In **A**, bars labeled with different letters are statistically different, same letters indicate that the values are not different statistically. * indicates statistical differences between marked bars **(B,C)** or between apocynin-treated and non-treated cultures **(D, E)**. Means were considered different if p<0.05, by two-way ANOVA test and Bonferroni post-test.

Phox KO macrophages showed reduced parasitism when compared with C57BL/6 WT macrophages. Our next step was to investigate if ROS were responsible for the lack of growth in Phox KO macrophages. Thus, we inhibited ROS production by C57BL/6 WT macrophages using different antioxidants. Pre-treatment with anti-oxidants was performed in order to ensure the status of the macrophage at the time of infection. The antioxidants SOD-PEG, CAT-PEG, NAC, and apocynin all reduced parasitism in C57BL/6 WT macrophages. This inhibition was more striking when we infected macrophages with TcMTH, since the levels of infection obtained with this parasite were greater and easily visualized after 48 hours (Fig 7A and 7B). When we observe the number of trypomastigotes released in the supernatant, the differences in infection are still more striking, for both TcWT and TcMTH. The number of trypomastigotes released in the supernatant of infected macrophages is reduced after treatment with apocynin (Fig 7E). The treatment of macrophages with NAC and apocynin also reduced the parasitism of the cells after infection with Y strain (Fig 7C and 7D).

### *T*. *cruzi* needs a signal provided by ROS to replicate efficiently

**Fig 8:**
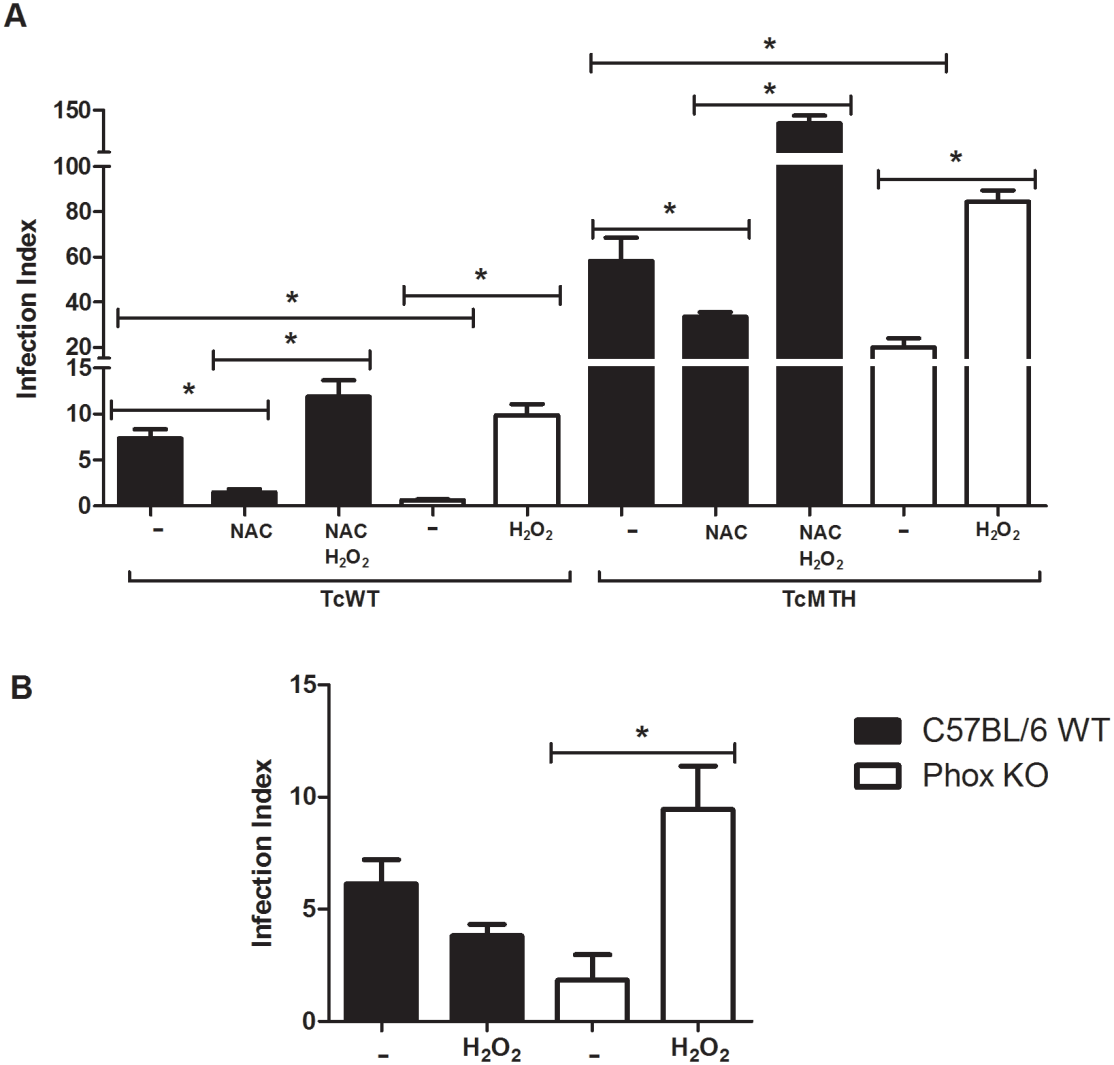
*T*. *cruzi* needs a signal provided by ROS to multiply efficiently. **(A)** Inflammatory macrophages obtained from Phox KO and C57BL/6 WT mice were incubated with 1mM of N-acetyl-cysteine (NAC) and 100μM H_2_O_2_ 2 hours before the infection with different parasites. Cells were washed to remove extracellular parasites and either fixed or re-incubated with medium or medium plus NAC or H_2_O_2_ for different times. The slides were stained and counted to determine the infection index. **(B)** Inflammatory macrophages from C57BL/6 WT and Phox KO mice were infected with CL Brenner strain of *T*. *cruzi* previously treated with 50μM H_2_O_2_ for 30 minutes. Cells were washed to remove extracellular parasites and either fixed or re-incubated with medium. The slides were stained and counted to determine the infection index. Data shown are representative one of three independent experiments performed in triplicate. A minimum of 200 macrophages were counted per group in triplicate. All data are presents as the means ± standard deviation. * indicates significant differences between marked bars or points, p<0.05, two-way ANOVA test with Bonferroni post-test.

Our results suggest that exposure to ROS promotes parasite replication. Some works have demonstrated that ROS could act as signal molecules to cells [11,36]. We propose that *T*. *cruzi* needs a signal provided by ROS produced by macrophages to thrive in this host cell. To test this hypothesis, we treated Phox KO macrophages with H_2_O_2_ before and after infection, and evaluated the infection index. We also treated NAC-treated C57BL/6 WT macrophages with H_2_O_2_. In both cases, the infection index was increased after treatment with H_2_O_2_ (Fig 8A). To clarify further this issue, we treated *T*. *cruzi* with H_2_O_2_ 30 minutes before infection. Our results show that parasites treated with H_2_O_2_ can infect Phox KO macrophages similarly to C57BL/6 WT macrophages (Fig 8B). In addition, treatment of parasites with H_2_O_2_ did not affect infection of C57BL/6 WT macrophages (Fig 8B). The results displayed in Fig 8A differ slightly from the ones presented in Fig 7, in that TcMTH parasites grew better in Phox KO macrophages than TcWT. This result was not repetitive, that is, in some experiments we observed this difference and in others we did not. The reason for the discrepancy is not clear to us at this point, but may be due to differences among parasite cultures obtained in different days. However, consistently parasites grew better in WT macrophages than in Phox KO macrophages, and TcMTH grew better than TcWT in WT machrophages.

### Lower concentrations of ROS promote parasite increase in macrophages and mice

**Fig 9:**
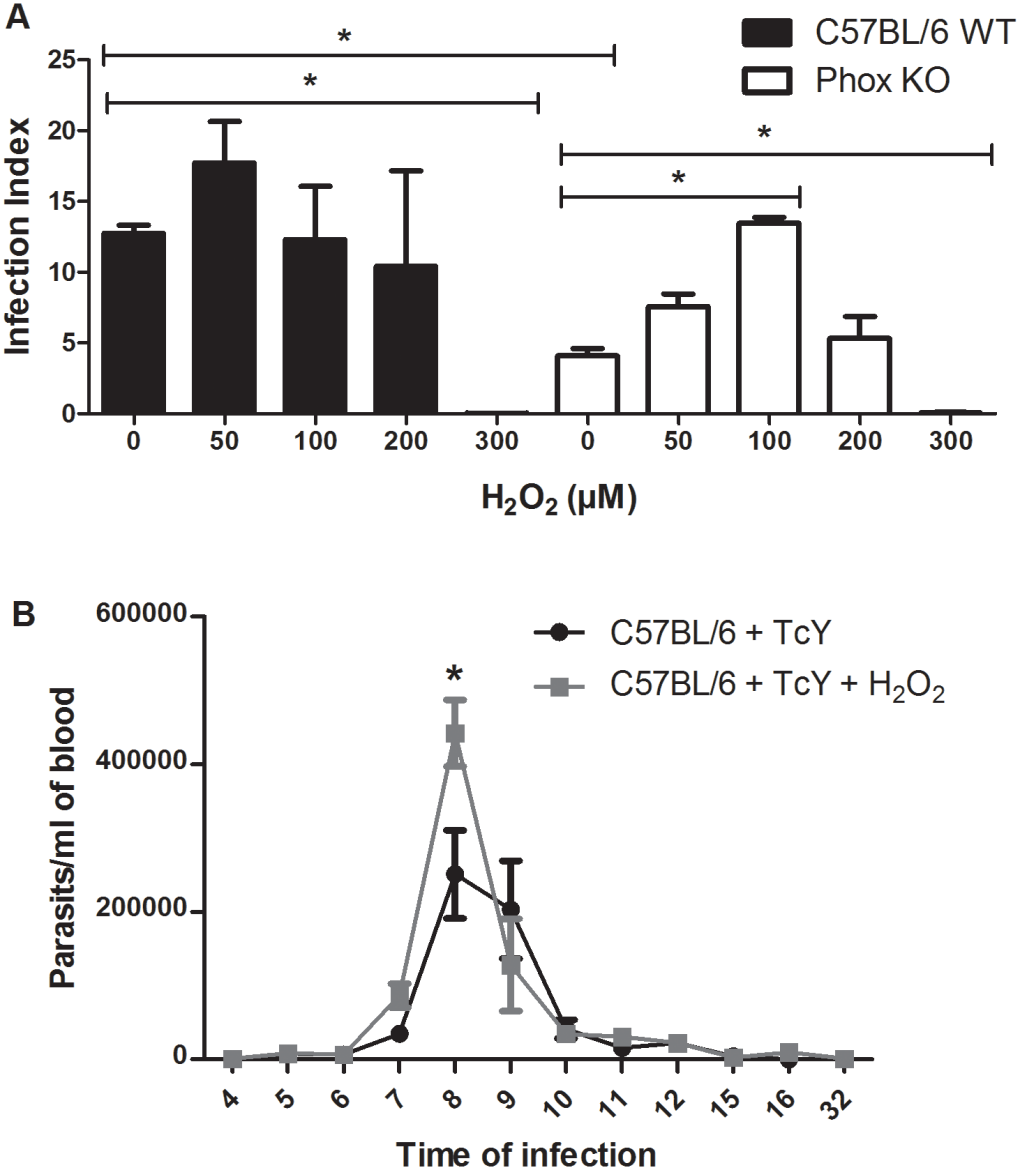
ROS in lower concentrations promote parasite increase in macrophages and mice. **(A)** Inflammatory macrophages obtained from Phox KO and C57BL/6 WT mice were infected with *T*. *cruzi* previously treated with different concentrations of H_2_O_2_ for 30 minutes. After 2 hours of infection, cells were washed to remove extracellular parasites and re-incubated with medium for 72 hours. The slides were stained and counted to determine the infection index. A minimum of 200 macrophages were counted per group in triplicate. Data shown are representative one of three independent experiments performed in triplicate. All data are presents as the means ± standard deviation. * indicates significant differences between marked bars or points, p<0.05, two-way ANOVA test with Bonferroni post-test. **(B)** C57BL/6 WT mice were infected with 1x10^5^ bloodstream parasites of Y strain treated or not with H_2_O_2_ for 30 minutes before infection. Parasitemia levels were evaluated (n = 5). Results are representative of three independent experiments, * indicates significant differences between points, p<0.05.

So far, our results seem paradoxical: oxidative-stress-resistant parasites multiplied better inside macrophages, but cells deficient in ROS production did not sustained *T*. *cruzi* infection. There is a broad response to oxidants in cells: after exposure to a relatively high concentration of ROS, oxidative stress damage generally occurs, while lower concentrations can exert important physiological roles in cellular signaling and proliferation [11]. Thus, we treated parasites with different concentrations of H_2_O_2_ and used these parasites to infect macrophages and mice. We found no differences in infection index of C57BL/6 macrophages using up to 200μM H_2_O_2_ (Fig 9A). However, in a higher concentration (300 μM) H_2_O_2_ was toxic to parasites. Lower concentrations of H_2_O_2_ (50μM and 100μM) promoted replication of parasites in Phox KO macrophages, while 200μM H_2_O_2_ brought the parasitism back down, and 300 μM H_2_O_2_ was also toxic to parasites in Phox KO macrophages. Our next step was to evaluate if the treatment of the parasite with H_2_O_2_ could affect *T*. *cruzi* capacity to infect mice. We treated blood trypomastigotes of the Y strain of *T*. *cruzi* with 100μM of H_2_O_2_ for 30 minutes, infected C57BL/6 WT mice by intraperitoneal injection of 10^3^ blood trypomastigotes and followed the course of infection. Our results indicate that C57BL/6 WT mice infected with treated parasites presented significantly higher parasitemia eight days after infection, compared with animals infected with control non treated parasites (Fig 9B). Our results suggest that the lower concentrations of ROS used contribute to growth of the parasite inside the cells, working like signaling molecules to the parasite.

### *In vivo* infections with genetically modified parasites

**Fig 10:**
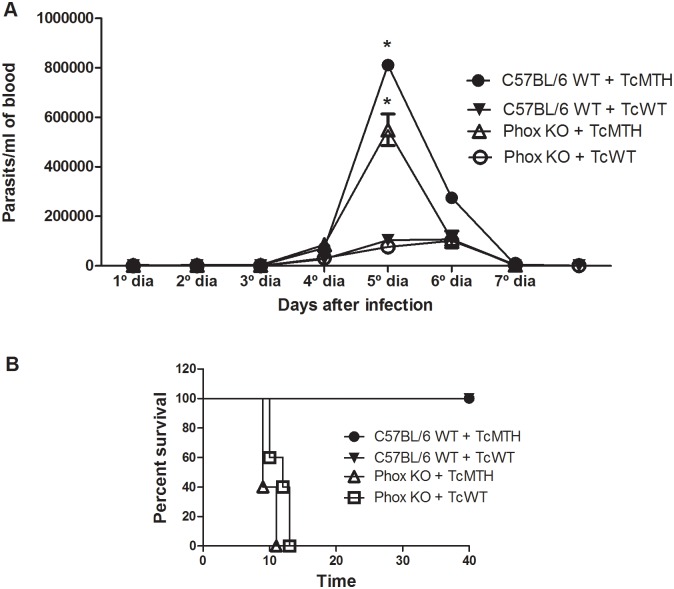
Parasitemia and mortality from C57BL/6 WT and Phox KO mice infected with TcWT and TcMTH parasites. Mice were infected into the peritoneum with 1x10^6^ parasites. **(A)** Mean parasitemia (n = 4) and **(B)** Mortality (n = 5). Results are representative of three independent experiments, * indicates significant differences between points, p<0.05.

To investigate whether the increase in the intracellular growth rate observed for the EcMutT parasites would also affect the course of infection *in vivo*, C57BL/6 WT and Phox KO mice were infected with one million TcWT or TcMTH, and parasitemia was evaluated from day 3 post-infection. The data obtained revealed that TcMTH-infected mice presented significantly higher parasitemia compared with animals infected with TcWT parasites and this difference was more prominent at 5 days post-infection (Fig 10A). This result corroborates the result obtained when the infection was performed in Swiss mice [29]. In addition, no difference in parasitemia was found between mouse strains. C57BL/6 WT and Phox KO mice displayed similar parasitemia, which peaked around 5 days post-infection (Fig 10A) and was subsequently controlled. However, mice deficient in functional NADPH oxidase do not survive infection (Fig 10B). While C57BL/6 WT mice presented 100% of survival after day 40 of infection, Phox KO animals exhibited high mortality when compared to C57BL/6 WT, starting at day 9 and reaching 100% mortality by 12 days of infection (Fig 10B and as previously published for the Y strain, [52]).

## Discussion

Oxidative stress, resultant from a deregulated ROS production, has been involved in pathogenesis of several diseases [53,53–55]. On the other hand, in higher eukaryotic cells, reactive oxygen species (ROS) recently emerged as important players in cellular signaling involved in cell growth and differentiation [56]. The regulated increase in free radicals in a temporary imbalance represents the physiological basis for redox regulation [57] and, in this case, ROS can act as secondary messengers in the intracellular signal transduction pathways [56,58,59]. In this paper, we show dual role for reactive oxygen species during infection with *T*. *cruzi*.

### ROS: Friend or foe?

In agreement with earlier observations in higher eukaryotes [60,61] some authors have pointed evidences for a role of ROS in growth and signaling events of pathogens [11,38,40–42]. In *Leishmania*, iron uptake controls H_2_O_2_ generation, which can act as a signaling molecule, initiating differentiation of promastigotes into infective amastigotes [43]. Sub-lethal doses of the superoxide-generating drug menadione and H_2_O_2_ results in increased resistance to H_2_O_2_ toxicity and increased virulence of *L*. *chagasi* promastigotes [62]. Corroborating these findings, the inhibition of ROS production by treatment with NAC reduced parasite burden in BALB/c mice infected with *Leishmania amazonensis* [63]. The exposure of *T*. *cruzi* to sub-lethal doses of H_2_O_2_ caused an increase in the level of antioxidant enzymes, and confers resistance to this oxidant [11,29]. Moreover, some authors have demonstrated different ROS-independent mechanisms used by cells to kill *T*. *cruzi*, contesting the necessity of these molecules in killing of parasites [64,65].

However, other studies relate ROS with the killing of parasites. Macrophages treated with phorbol myristate acetate (PMA), which triggers respiratory burst, are incapable of releasing ROS upon subsequent re-stimulation. In these cells, PMA pre-treatment contributes to growth of *T*. *cruzi*, pointing to the importance of a respiratory burst mechanism in killing of intracellular parasites [66]. In another work, the ability to release H_2_O_2_ and the ability to kill trypanosomes were correlated in macrophages [67] and strong evidence for peroxynitrite as a mediator of *T*. *cruzi* killing was also found [4]. Using a variant clone derived from the cloned macrophage cell line J774, which lacked the capacity of producing ROS, Tanaka *et al*. demonstrated that *T*. *cruzi* grew better in this variant cell line [33] and that H_2_O_2_ is associated with the killing of parasites [68].

Hence, from the exposed above, ROS may be friend to the parasite or foe.

### ROS: Foe

*T*. *cruzi* is exposed to oxidative stress conditions in its life cycle [2,10,69] and this may generate oxidized nucleotides, causing DNA damage. We had already demonstrated that parasites with enhanced 8-oxo-dGTPase activity multiply better than wild type parasites in Swiss mice [29]. In the present work, we show that, although both wild-type and recombinant parasites had the same capacity of invading macrophages, modified parasites presented improved growth in macrophage cultures and confirm that these parasites multiply better *in vivo*, using C57BL6/WT mice, a different animal model. Hence, protection of DNA against oxidative stress is beneficial to the parasite performance both *in vivo* and *in vitro*. The reason for this better performance may be as simple as the quicker replication when there is less necessity of DNA repair, or a more complex mechanism involving increased expression of protective enzymes, as discussed below, and which exact cause is currently unknown.

The hydrolysis of 8-oxo-dGTP could prevent DNA lesions and this could explain the greater replicative capacity of parasites with enhanced 8-oxo-dGTPase activity. Indeed, we demonstrated that modified parasites prevent 8-oxo-dGTP incorporation into DNA when exposed to H_2_O_2_. Furthermore, TcMTH and EcMutT parasites expressed more TcCPx and TcMPx after exposure to H_2_O_2_ than WT parasites [29] and, importantly, incorporate less peroxynitrite (Fig 3). The enzymes TcCPX and TcMPX have the capacity to detoxify ONOO^−^, H_2_O_2_ and small-chain organic hydroperoxidases [18,21], which would explain the smaller concentrations of peroxynitrite inside TcMTH parasites compared to TcWT. Thus, these latter set of data speak for a more complex reason for higher proliferation of TcMTH parasites, which could involve increased expression of antioxidant enzymes.

*T*. *cruzi* contains four iron superoxide dismutases (FeSODs) that eliminate superoxide radicals by dismutation into H_2_O_2_ and molecular oxygen [70]. The levels of cytosolic SODB did not increase after oxidative treatment with H_2_O_2_, possibly because this enzyme is not involved with H_2_O_2_ detoxification. Furthermore, there are no differences in the levels of SODB between modified and wild type parasites. *T*. *cruzi* cytosolic FeSODB is particularly resistant to peroxinitrite inactivation, suggesting it participates mainly as an antioxidant defense enzyme, while mitochondrial FeSODA may act as an oxidative stress sensor participating in O_2_^●−^-mediated redox process of cell signaling [21,71]. Some works show that levels of TcCPX, TcMPX and mitochondrial SODA are up-regulated in infective forms of the parasite [20,72,73]. The relationship of these enzymes with the infective capacity of the parasite further helps to explain why modified parasites replicate more successfully *in vitro* and *in vivo*.

Thus, the results obtained here reinforce the idea that ROS are deleterious to *T*. *cruzi*, since modified parasites grow better, probably because they are better able to deal with oxidative stress conditions.

### ROS: Friend

Phox KO macrophages infected with CL Brenner strain of *T*. *cruzi* had decreased parasitism compared to C57BL/6 WT macrophages. This difference is not related to the uptake of parasites, since our results demonstrate that both macrophages presented the same parasite uptake. Similarly, knockout mice to p47 subunit of NADPH oxidase (p47^Phox KO^) were not compromised in parasite uptake capacity [74]. We also show that the treatment with antioxidants reduce parasite replication. These results indicate that the parasite needs a signal provided by macrophages to replicate efficiently within these cells. These data are in agreement with a work published by Paiva *et al* [35] using Y strain of *T*. *cruzi*, but are in contrast with work published by Dhiman and Garg [74] using Sylvio X10/4 strain and p47^Phox KO^ mice. This latter work shows no differences in the number of trypomastigotes released in the supernatants of infected macrophages from p47^Phox KO^ and WT mice [74]. This difference could be related to the type of strain used or to levels of ROS detected in macrophages after parasite infection. Our results show that *T*. *cruzi* infection did not induce alterations in ROS levels detected in Phox KO macrophages, which are similar to levels observed in resting cells. The same result was obtained when we used zymosan as a stimulus (S4 Fig). Dhiman and Garg, however, showed that ROS levels detected in p47^Phox KO^ cells are reduced when compared to levels produced by WT cells, but are significantly higher when compared to basal levels of production in non-infected cells [74].

Our results suggest that the parasite needs a signal given by ROS in order to grow inside the cells. It is unlikely that O_2_^●−^ is the reactive oxygen species responsible for signaling in *T*. *cruzi*, because of the anionic nature and restricted capacity of this molecule in to cross membranes. So, the exposure of parasites in the cytosol to this radical would be unlikely. On the other hand, H_2_O_2_ is an oxidant with higher diffusional capacity. Although H_2_O_2_ is known for its cytotoxic effects, recently it has emerged as an important regulator of signal transduction in eukaryotic cells. This positive (signaling) or negative (damage) effect is dependent of the level of H_2_O_2_ and of the cell type under investigation [75]. We show here that parasite growth in Phox KO macrophages could be triggered if we treated Phox KO macrophages or antioxidant treated-WT macrophages with H_2_O_2_ before infection. Treatment of parasites with H_2_O_2_ before infection also induced the recovery of replicative capacity in Phox KO macrophages. In addition, treatment of blood-derived parasites with H_2_O_2_ used for in vivo infection increased parasitemia levels in C57BL/6 WT mice. Although the mechanism by which ROS promotes parasite proliferation remains to be elucidated, our data suggest that this signal is given to the parasite instead the macrophage, since pre-treatment of parasites is sufficient to promote growth in Phox KO macrophages and *in vivo*.

Further evidence that H_2_O_2_ is the signal for parasite replication is that treatment of macrophages with catalase, an enzyme that promotes H_2_O_2_ detoxification, also reduced parasitism. This signal could be provide by a direct or an indirect effect of H_2_O_2_. Removal of H_2_O_2_ by antioxidant enzymes prevents H_2_O_2_ signaling, but recent studies have identified several peroxide-signaling mechanisms in which antioxidant enzymes act as H_2_O_2_ sensors. The high affinity of some peroxidases for H_2_O_2_ makes them suited for hydrogen peroxide-sensing [75]. The peroxidase class of H_2_O_2_-scavenging enzymes has conserved cysteine residues in their catalytic sites, which are targets for oxidation by H_2_O_2_ [76]. The initial H_2_O_2_ sensing event would be the oxidation of an antioxidant enzyme, which then leads to changes in the activity of associated components of the signaling pathway [75]. A recent work shows that H_2_O_2_ signaling could be sensed by cysteine-containing proteins, such as thiol peroxidase peroxiredoxin-2 (PRX-2), which would become oxidized and would transmit oxidative equivalents to the redox-regulated transcription factor STAT3. Prx2 catalyzes the formation of disulfide-linked STAT3 oligomers, which compromises its capacity to promote transcription [77]. This could influence the cellular response, activating or inhibiting different cell pathways related with pathogen invasion.

Some studies have suggested that oxidative stress is important for *T*. *cruzi* proliferation [35,36]. One evidence for the role of ROS in signaling events is that ROS or heme-induced ROS activate a CaM Kinase II-like pathway muttriggering the proliferation of the epimastigote forms of *T*. *cruzi* [36]. In addition, the oxidative stress generated in response to Y strain of *T*. *cruzi* contributes to the maintenance of high parasite burdens in macrophages [35]. The treatment with antioxidants inhibited epimastigote proliferation *in vitro* [36] and reduced *T*. *cruzi* parasitemia [78].

Once in the vertebrate host, trypomastigotes invade cells at the inoculation site (e.g., fibroblasts, macrophages, and epithelial cells) [79,80]. *T*. *cruzi* multiplies inside resident macrophages and disrupts these cells, which release infective trypomastigote forms that reach blood circulation and disseminates to other cells, like myocardium and autonomic nervous system ganglion cells that innervate esophagus and intestine walls. We found no differences in parasitemia in Phox KO and C57BL/6 WT mice. This could be because ROS are important to signaling events in macrophages, but not in other host cells, like for example fibroblasts [35]. These data are in contrast with the reported increased parasite burden in apocynin-treated mice and in p47^Phox KO^ mice infected with strain Sylvio X10/4 [74,81] and also in contrast with the reported reduced parasitemia in Phox KO mice infected with Y strain [35]. Data with p47^Phox KO^ mice show that these mice succumbed to infection with SylvioX10/4 strain probably because of a compromised CD8^+^T cell response, leading to increased parasite burden and pathogenesis [74]. Our results on *in vivo* infection with CL Brenner strain are in agreement with the previously published work by our group with Y strain [52]. In that paper, we showed that infected Phox KO mice succumb to infection probably due to low blood pressure caused by excess ^●^NO, which was not quenched by superoxide. Apocynin treatment increased parasitemia in C3H/HeN mice infected with the SylvioX10/4 strain. The reason for the discrepancy between data obtained in Phox KO mice in our hands and apocynin-treated mice described earlier [74,81] may be several, including mouse strain and parasite strain. The SylvioX10/4 strain grows more slowly in mice [74,81], and it is not clear if parasitemia in apocynin-treated mice was determined in the acute or in the chronic phase of infection. In addition, it is possible that apocynin inhibits other oxidative mechanisms independent of NOX2. *In vitro*, apocynin has been shown to have an oxidative effect [82]. In our hands, apocynin effects were consistent with the inhibition of NOX2 in macrophages.

The exact mechanism by which low oxidant production enhances *T*. *cruzi* infection remains to be elucidated. One possibility is that ROS could generate the oxidation of 8-oxoG, resulting in the monophosphate form, 8-oxodGMP, which could be acting as a second messenger to the cell, indicating the presence of oxidative stress and preparing the parasite to be more resistant. This is currently under investigation.

### Conclusion

In the present study we attempted to clarify the importance of ROS in *T*. *cruzi* infections. We found that modified parasites, more resistant to DNA damage by ROS, multiply better inside macrophages, but their proliferation, as well as the proliferation of wild-type parasites, is significantly reduced when ROS production is inhibited in the host cell. A possible explanation is that parasites need minimal levels of ROS, which would work as a signal for replication. However, high levels of ROS are deleterious to the parasite, inducing, for example, DNA damage. In this way, parasites over-expressing 8-oxo-GTPase could be more fit (since less DNA repair is necessary, for instance) and escape from the negative effects induced by ROS, by decreasing double-strand breaks and thus lethal lesions, increasing their replicative capacity.

## Supporting Information

S1 Fig Replicate of the experiment presented in Fig 2: Growth of parasites with enhanced 8-oxod-GTP pyrophosphohydrolase activity in macrophages. Inflammatory macrophages obtained from peritoneal cavity of C57BL/6 WT mice were infected with wild type and modified parasites. The cells were washed to remove extracellular parasites and either fixed or re-incubated with medium for different times. **(A)** Number of parasites per macrophage. **(B)** Number of infected macrophage per total macrophage. **(C)** Infection index for each parasite population. **(D)** Number of parasites released into the macrophage culture supernatant between the third and seventh day after infection. Data shown are representative of a second of three independent experiments performed in triplicate (cells were pooled from three mice for each replicate). All data are presented as the means ± standard deviation. * indicates significant differences between marked bars or between points, p<0.05, two-way ANOVA test with Bonferroni post-test.
(TIF)

S2 Fig Representative western blot of experiment presented in Fig 3B: CPX expression in TcWT and TcMTH epimastigotes. Cytosolic tryparedoxin peroxidase in parasite extracts was detected by Western Blot using an anti-TcCPX specific antibody. Western blot analysis of CPx from non-treated TcWT (1) and EcMutT (3), or 200 μM H_2_O_2_-treated TcWT (2) and EcMutT (4) parasites. Cruzipain was used as loading control. The signal intensity obtained for CPX enzyme in parasites was set according to the cruzipain quantity verified.
(TIF)

S3 Fig Cell viability assay of epimastigotes treated with different concentrations of H_2_O_2_. *T*. *cruzi* epimastigote viability in the presence of different concentrations of H_2_O_2_ was evaluated by the quantitative colorimetric MTT [3-(4,5-dimethylthiazol-2-yl)-2,5-diphenyl tetrazolium bromide)] assay. Cells (5 x10^6^/mL) were incubated in a 96-well plate with H_2_O_2_ for 30 minutes, washed and MTT added (final concentration, 1 mg/mL). After a 4-h incubation, DMSO was added to dissolve the formazan crystals and absorbance measured at 590 nm. Parasite viability (%) was calculated regarding the control. We used 1% Triton X-100 as a positive control.
(TIF)

S4 Fig Production of reactive oxygen species by macrophages stimulated with Y strain of *T*. *cruzi* and zymosan. Thioglycolate-elicited macrophages were harvested from the peritoneal cavity of C57BL/6 WT and Phox KO mice 4 days after stimulation. Reactive oxygen species production by macrophages was detected by luminol. Chemiluminescence was continuously measured immediately after *T*. *cruzi* (TcY) or zymosan (Z, 1x10^7^U/well) addition to the macrophage monolayer, and the area under the obtained curves was calculated. The graphs are representative of three independent experiments performed in triplicate (cells were pooled from three mice for each replicate). Bars marked by different letters are statistically different (p<0.05, one-way ANOVA test with Bonferroni post-test).
(TIF)

## References

[1] Kierszenbaum F, Knecht E, Budzko DB, Pizzimenti MC. Phagocytosis: a defense mechanism against infection with *Trypanosoma cruzi*. J Immunol. 1974; 112: 1839–1844 4361979

[2] Cardoni RL, Antunez MI, Morales C, Nantes IR. Release of reactive oxygen species by phagocytic cells in response to live parasites in mice infected with *Trypanosoma cruzi*. Am J Trop Med Hyg. 1997; 56: 329–334 9129538 10.4269/ajtmh.1997.56.329

[3] Melo RC, Fabrino DL, D'Avila H, Teixeira HC, Ferreira AP. Production of hydrogen peroxide by peripheral blood monocytes and specific macrophages during experimental infection with *Trypanosoma cruzi* in vivo. Cell Biol Int. 2003; 27: 853–861 14499666 10.1016/s1065-6995(03)00173-2

[4] Alvarez MN, Peluffo G, Piacenza L, Radi R. Intraphagosomal peroxynitrite as a macrophage-derived cytotoxin against internalized *Trypanosoma cruzi*: consequences for oxidative killing and role of microbial peroxiredoxins in infectivity. J Biol Chem. 2011; 286: 6627–6640 10.1074/jbc.M110.167247 21098483 PMC3057850

[5] Babior BM. The respiratory burst of phagocytes. J Clin Invest. 1984; 73: 599–601 6323522 10.1172/JCI111249 PMC425058

[6] Groemping Y, Rittinger K. Activation and assembly of the NADPH oxidase: a structural perspective. Biochem J. 2005; 386: 401–416 15588255 10.1042/BJ20041835 PMC1134858

[7] Alvarez MN, Piacenza L, Irigoin F, Peluffo G, Radi R. Macrophage-derived peroxynitrite diffusion and toxicity to *Trypanosoma cruzi*. Arch Biochem Biophys. 2004; 432: 222–232 15542061 10.1016/j.abb.2004.09.015

[8] Piacenza L, Peluffo G, Alvarez MN, Kelly JM, Wilkinson SR, Radi R. Peroxiredoxins play a major role in protecting *Trypanosoma cruzi* against macrophage- and endogenously-derived peroxynitrite. Biochem J. 2008; 410: 359–368 17973627 10.1042/BJ20071138 PMC2441817

[9] Krauth-Siegel RL, Comini MA. Redox control in trypanosomatids, parasitic protozoa with trypanothione-based thiol metabolism. Biochim Biophys Acta. 2008; 1780: 1236–1248 10.1016/j.bbagen.2008.03.006 18395526

[10] Piacenza L, Alvarez MN, Peluffo G, Radi R. Fighting the oxidative assault: the *Trypanosoma cruzi* journey to infection. Curr Opin Microbiol. 2009; 12: 415–421 10.1016/j.mib.2009.06.011 19616990

[11] Finzi JK, Chiavegatto CW, Corat KF, Lopez JA, Cabrera OG, Mielniczki-Pereira AA, et al *Trypanosoma cruzi* response to the oxidative stress generated by hydrogen peroxide. Mol Biochem Parasitol. 2004; 133: 37–43 14668010 10.1016/j.molbiopara.2003.08.011

[12] Shames SL, Fairlamb AH, Cerami A, Walsh CT. Purification and characterization of trypanothione reductase from *Crithidia fasciculata*, a newly discovered member of the family of disulfide-containing flavoprotein reductases. Biochemistry. 1986; 25: 3519–3526 3718941 10.1021/bi00360a007

[13] Wilkinson SR, Meyer DJ, Kelly JM. Biochemical characterization of a trypanosome enzyme with glutathione-dependent peroxidase activity. Biochem J. 2000; 352 Pt 3: 755–761 11104683 PMC1221514

[14] Wilkinson SR, Temperton NJ, Mondragon A, Kelly JM. Distinct mitochondrial and cytosolic enzymes mediate trypanothione-dependent peroxide metabolism in *Trypanosoma cruzi*. J Biol Chem. 2000; 275: 8220–8225 10713147 10.1074/jbc.275.11.8220

[15] Wilkinson SR, Taylor MC, Touitha S, Mauricio IL, Meyer DJ, Kelly JM. TcGPXII, a glutathione-dependent *Trypanosoma cruzi* peroxidase with substrate specificity restricted to fatty acid and phospholipid hydroperoxides, is localized to the endoplasmic reticulum. Biochem J. 2002; 364: 787–794 12049643 10.1042/BJ20020038 PMC1222628

[16] Wilkinson SR, Meyer DJ, Taylor MC, Bromley EV, Miles MA, Kelly JM. The *Trypanosoma cruzi* enzyme TcGPXI is a glycosomal peroxidase and can be linked to trypanothione reduction by glutathione or tryparedoxin. J Biol Chem. 2002; 277: 17062–17071 11842085 10.1074/jbc.M111126200

[17] Wilkinson SR, Obado SO, Mauricio IL, Kelly JM. *Trypanosoma cruzi* expresses a plant-like ascorbate-dependent hemoperoxidase localized to the endoplasmic reticulum. Proc Natl Acad Sci U S A. 2002; 99: 13453–13458 12351682 10.1073/pnas.202422899 PMC129694

[18] Trujillo M, Budde H, Pineyro MD, Stehr M, Robello C, Flohe L, et al *Trypanosoma brucei* and *Trypanosoma cruzi* tryparedoxin peroxidases catalytically detoxify peroxynitrite via oxidation of fast reacting thiols. J Biol Chem. 2004; 279: 34175–34182 15155760 10.1074/jbc.M404317200

[19] Wilkinson SR, Obado SO, Mauricio IL, Kelly JM. *Trypanosoma cruzi* expresses a plant-like ascorbate-dependent hemoperoxidase localized to the endoplasmic reticulum. Proc Natl Acad Sci U S A. 2002; 99: 13453–13458 12351682 10.1073/pnas.202422899 PMC129694

[20] Piacenza L, Zago MP, Peluffo G, Alvarez MN, Basombrio MA, Radi R. Enzymes of the antioxidant network as novel determiners of *Trypanosoma cruzi* virulence. Int J Parasitol. 2009; 39: 1455–1464 10.1016/j.ijpara.2009.05.010 19505468 PMC3909716

[21] Piacenza L, Peluffo G, Alvarez MN, Martinez A, Radi R. *Trypanosoma cruzi* antioxidant enzymes as virulence factors in Chagas disease. Antioxid Redox Signal. 2013; 19: 723–734 10.1089/ars.2012.4618 22458250 PMC3739954

[22] Peloso EF, Vitor SC, Ribeiro LH, Pineyro MD, Robello C, Gadelha FR. Role of *Trypanosoma cruzi* peroxiredoxins in mitochondrial bioenergetics. J Bioenerg Biomembr. 2011; 43: 419–424 10.1007/s10863-011-9365-4 21732175

[23] Neeley WL, Essigmann JM. Mechanisms of formation, genotoxicity, and mutation of guanine oxidation products. Chem Res Toxicol. 2006; 19: 491–505 16608160 10.1021/tx0600043

[24] Cheng KC, Cahill DS, Kasai H, Nishimura S, Loeb LA. 8-Hydroxyguanine, an abundant form of oxidative DNA damage, causes G——T and A——C substitutions. J Biol Chem. 1992; 267: 166–172 1730583

[25] Hsu GW, Ober M, Carell T, Beese LS. Error-prone replication of oxidatively damaged DNA by a high-fidelity DNA polymerase. Nature. 2004; 431: 217–221 15322558 10.1038/nature02908

[26] Michaels ML, Miller JH. The GO system protects organisms from the mutagenic effect of the spontaneous lesion 8-hydroxyguanine (7,8-dihydro-8-oxoguanine). J Bacteriol. 1992; 174: 6321–6325 1328155 10.1128/jb.174.20.6321-6325.1992 PMC207574

[27] Barnes DE, Lindahl T. Repair and genetic consequences of endogenous DNA base damage in mammalian cells. Annu Rev Genet. 2004; 38: 445–476 15568983 10.1146/annurev.genet.38.072902.092448

[28] El-Sayed NM, Myler PJ, Bartholomeu DC, Nilsson D, Aggarwal G, Tran AN, et al The genome sequence of *Trypanosoma cruzi*, etiologic agent of Chagas disease. Science. 2005; 309: 409–415 16020725 10.1126/science.1112631

[29] Aguiar PH, Furtado C, Repoles BM, Ribeiro GA, Mendes IC, Peloso EF, et al Oxidative stress and DNA lesions: the role of 8-oxoguanine lesions in *Trypanosoma cruzi* cell viability. PLoS Negl Trop Dis. 2013; 7: e2279 10.1371/journal.pntd.0002279 23785540 PMC3681716

[30] Mildvan AS, Weber DJ, Abeygunawardana C. Solution structure and mechanism of the MutT pyrophosphohydrolase. Adv Enzymol Relat Areas Mol Biol. 1999; 73: 183–207 10218109 10.1002/9780470123195.ch6

[31] Abeygunawardana C, Weber DJ, Gittis AG, Frick DN, Lin J, Miller AF, et al Solution structure of the MutT enzyme, a nucleoside triphosphate pyrophosphohydrolase. Biochemistry. 1995; 34: 14997–15005 7578113 10.1021/bi00046a006

[32] Paiva CN, Bozza MT. Are reactive oxygen species always detrimental to pathogens? Antioxid Redox Signal. 2014; 20: 1000–1037 10.1089/ars.2013.5447 23992156 PMC3924804

[33] Tanaka Y, Tanowitz H, Bloom BR. Growth of *Trypanosoma cruzi* in a cloned macrophage cell line and in a variant defective in oxygen metabolism. Infect Immun. 1983; 41: 1322–1331 6350185 10.1128/iai.41.3.1322-1331.1983 PMC264642

[34] Andrews NW. Oxidative stress and intracellular infections: more iron to the fire. J Clin Invest. 2012; 122: 2352–2354 10.1172/JCI64239 22728929 PMC3386837

[35] Paiva CN, Feijo DF, Dutra FF, Carneiro VC, Freitas GB, Alves LS, et al Oxidative stress fuels *Trypanosoma cruzi* infection in mice. J Clin Invest. 2012; 122: 2531–2542 10.1172/JCI58525 22728935 PMC3386808

[36] Nogueira NP, de Souza CF, Saraiva FM, Sultano PE, Dalmau SR, Bruno RE, et al Heme-induced ROS in *Trypanosoma cruzi* activates CaMKII-like that triggers epimastigote proliferation. One helpful effect of ROS. PLoS One. 2011; 6: e25935 10.1371/journal.pone.0025935 22022475 PMC3191175

[37] Jung JY, Madan-Lala R, Georgieva M, Rengarajan J, Sohaskey CD, Bange FC, et al The intracellular environment of human macrophages that produce nitric oxide promotes growth of mycobacteria. Infect Immun. 2013; 81: 3198–3209 10.1128/IAI.00611-13 23774601 PMC3754229

[38] Oberley-Deegan RE, Rebits BW, Weaver MR, Tollefson AK, Bai X, McGibney M, et al An oxidative environment promotes growth of *Mycobacterium abscessus*. Free Radic Biol Med. 2010; 49: 1666–1673 10.1016/j.freeradbiomed.2010.08.026 20807564 PMC2970643

[39] Jimenez-Lopez C, Collette JR, Brothers KM, Shepardson KM, Cramer RA, Wheeler RT, et al *Candida albicans* induces arginine biosynthetic genes in response to host-derived reactive oxygen species. Eukaryot Cell. 2013; 12: 91–100 10.1128/EC.00290-12 23143683 PMC3535846

[40] Fraternale A, Paoletti MF, Casabianca A, Nencioni L, Garaci E, Palamara AT, et al GSH and analogs in antiviral therapy. Mol Aspects Med. 2009; 30: 99–110 10.1016/j.mam.2008.09.001 18926849

[41] Tung WH, Hsieh HL, Yang CM. Enterovirus 71 induces COX-2 expression via MAPKs, NF-kappaB, and AP-1 in SK-N-SH cells: Role of PGE(2) in viral replication. Cell Signal. 2010; 22: 234–246 10.1016/j.cellsig.2009.09.018 19800403

[42] Vlahos R, Stambas J, Bozinovski S, Broughton BR, Drummond GR, Selemidis S. Inhibition of Nox2 oxidase activity ameliorates influenza A virus-induced lung inflammation. PLoS Pathog. 2011; 7: e1001271 10.1371/journal.ppat.1001271 21304882 PMC3033375

[43] Mittra B, Cortez M, Haydock A, Ramasamy G, Myler PJ, Andrews NW. Iron uptake controls the generation of *Leishmania* infective forms through regulation of ROS levels. J Exp Med. 2013; 210: 401–416 10.1084/jem.20121368 23382545 PMC3570109

[44] Pollock JD, Williams DA, Gifford MA, Li LL, Du X, Fisherman J, et al Mouse model of X-linked chronic granulomatous disease, an inherited defect in phagocyte superoxide production. Nat Genet. 1995; 9: 202–209 7719350 10.1038/ng0295-202

[45] DaRocha WD, Silva RA, Bartholomeu DC, Pires SF, Freitas JM, Macedo AM, et al Expression of exogenous genes in *Trypanosoma cruzi*: improving vectors and electroporation protocols. Parasitol Res. 2004; 92: 113–120 14634799 10.1007/s00436-003-1004-5

[46] Dalton DK, Pitts-Meek S, Keshav S, Figari IS, Bradley A, Stewart TA. Multiple defects of immune cell function in mice with disrupted interferon-gamma genes. Science. 1993; 259: 1739–1742 8456300 10.1126/science.8456300

[47] Green LC, Wagner DA, Glogowski J, Skipper PL, Wishnok JS, Tannenbaum SR. Analysis of nitrate, nitrite, and [15N]nitrate in biological fluids. Anal Biochem. 1982; 126: 131–138 7181105 10.1016/0003-2697(82)90118-x

[48] Moore MK, Viselli SM. Staining and quantification of proteins transferred to polyvinylidene fluoride membranes. Anal Biochem. 2000; 279: 241–242 10706793 10.1006/abio.2000.4482

[49] Struthers L, Patel R, Clark J, Thomas S. Direct detection of 8-oxodeoxyguanosine and 8-oxoguanine by avidin and its analogues. Anal Biochem. 1998; 255: 20–31 9448838 10.1006/abio.1997.2354

[50] Gad H, Koolmeister T, Jemth AS, Eshtad S, Jacques SA, Strom CE, et al MTH1 inhibition eradicates cancer by preventing sanitation of the dNTP pool. Nature. 2014; 508: 215–221 10.1038/nature13181 24695224

[51] BRENER Z. Therapeutic activity and criterion of cure on mice experimentally infected with *Trypanosoma cruzi*. Rev Inst Med Trop Sao Paulo. 1962; 4: 389–396 14015230

[52] Santiago HC, Gonzalez Lombana CZ, Macedo JP, Utsch L, Tafuri WL, Campagnole-Santos MJ, et al NADPH phagocyte oxidase knockout mice control *Trypanosoma cruzi* proliferation, but develop circulatory collapse and succumb to infection. PLoS Negl Trop Dis. 2012; 6: e1492 10.1371/journal.pntd.0001492 22348160 PMC3279332

[53] Dreher D, Junod AF. Role of oxygen free radicals in cancer development. Eur J Cancer. 1996; 32A: 30–38 8695238 10.1016/0959-8049(95)00531-5

[54] Houstis N, Rosen ED, Lander ES. Reactive oxygen species have a causal role in multiple forms of insulin resistance. Nature. 2006; 440: 944–948 16612386 10.1038/nature04634

[55] Singal PK, Khaper N, Farahmand F, Bello-Klein A. Oxidative stress in congestive heart failure. Curr Cardiol Rep. 2000; 2: 206–211 10980894 10.1007/s11886-000-0070-x

[56] Droge W. Free radicals in the physiological control of cell function. Physiol Rev. 2002; 82: 47–95 11773609 10.1152/physrev.00018.2001

[57] Frein D, Schildknecht S, Bachschmid M, Ullrich V. Redox regulation: a new challenge for pharmacology. Biochem Pharmacol. 2005; 70: 811–823 15899473 10.1016/j.bcp.2005.04.012

[58] Rudolph TK, Freeman BA. Transduction of redox signaling by electrophile-protein reactions. Sci Signal. 2009; 2: re7 19797270 10.1126/scisignal.290re7 PMC4106464

[59] Trachootham D, Lu W, Ogasawara MA, Nilsa RD, Huang P. Redox regulation of cell survival. Antioxid Redox Signal. 2008; 10: 1343–1374 10.1089/ars.2007.1957 18522489 PMC2932530

[60] Rhee SG. Cell signaling. H_2_O_2_, a necessary evil for cell signaling. Science. 2006; 312: 1882–1883 16809515 10.1126/science.1130481

[61] Sarsour EH, Kumar MG, Chaudhuri L, Kalen AL, Goswami PC. Redox control of the cell cycle in health and disease. Antioxid Redox Signal. 2009; 11: 2985–3011 10.1089/ARS.2009.2513 19505186 PMC2783918

[62] Wilson ME, Andersen KA, Britigan BE. Response of *Leishmania chagasi* promastigotes to oxidant stress. Infect Immun. 1994; 62: 5133–5141 7927797 10.1128/iai.62.11.5133-5141.1994 PMC303235

[63] Monteiro MC, Marques FC, Blazius RD, Santos da SO, de Queiroz CF, Bento DB, et al N-acetyl-L: -cysteine reduces the parasitism of BALB/c mice infected with *Leishmania amazonensis*. Parasitol Res. 2008; 102: 801–803 18094999 10.1007/s00436-007-0827-x

[64] McCabe RE, Mullins BT. Failure of *Trypanosoma cruzi* to trigger the respiratory burst of activated macrophages. Mechanism for immune evasion and importance of oxygen-independent killing. J Immunol. 1990; 144: 2384–2388 2155965

[65] Munoz-Fernandez MA, Fernandez MA, Fresno M. Activation of human macrophages for the killing of intracellular *Trypanosoma cruzi* by TNF-alpha and IFN-gamma through a nitric oxide-dependent mechanism. Immunol Lett. 1992; 33: 35–40 1330900 10.1016/0165-2478(92)90090-b

[66] Murray HW. Pretreatment with phorbol myristate acetate inhibits macrophage activity against intracellular protozoa. J Reticuloendothel Soc. 1982; 31: 479–487 6288939

[67] Nathan C, Nogueira N, Juangbhanich C, Ellis J, Cohn Z. Activation of macrophages in vivo and in vitro. Correlation between hydrogen peroxide release and killing of *Trypanosoma cruzi*. J Exp Med. 1979; 149: 1056–1068 376774 10.1084/jem.149.5.1056 PMC2184875

[68] Tanaka Y, Kiyotaki C, Tanowitz H, Bloom BR. Reconstitution of a variant macrophage cell line defective in oxygen metabolism with a H_2_O_2_-generating system. Proc Natl Acad Sci U S A. 1982; 79: 2584–2588 7045866 10.1073/pnas.79.8.2584 PMC346244

[69] Paes MC, Oliveira MB, Oliveira PL. Hydrogen peroxide detoxification in the midgut of the blood-sucking insect, *Rhodnius prolixus*. Arch Insect Biochem Physiol. 2001; 48: 63–71 11568965 10.1002/arch.1058

[70] Mateo H, Marin C, Perez-Cordon G, Sanchez-Moreno M. Purification and biochemical characterization of four iron superoxide dismutases in *Trypanosoma cruzi*. Mem Inst Oswaldo Cruz. 2008; 103: 271–276 18592100 10.1590/s0074-02762008000300008

[71] Prolo C, Alvarez MN, Radi R. Peroxynitrite, a potent macrophage-derived oxidizing cytotoxin to combat invading pathogens. Biofactors. 2014; 40: 215–225 10.1002/biof.1150 24281946 PMC3997626

[72] Atwood JA III, Weatherly DB, Minning TA, Bundy B, Cavola C, Opperdoes FR, et al The *Trypanosoma cruzi* proteome. Science. 2005; 309: 473–476 16020736 10.1126/science.1110289

[73] Parodi-Talice A, Monteiro-Goes V, Arrambide N, Avila AR, Duran R, Correa A, et al Proteomic analysis of metacyclic trypomastigotes undergoing *Trypanosoma cruzi* metacyclogenesis. J Mass Spectrom. 2007; 42: 1422–1432 17960573 10.1002/jms.1267

[74] Dhiman M, Garg NJ. P47phox-/- mice are compromised in expansion and activation of CD8+ T cells and susceptible to *Trypanosoma cruzi* infection. PLoS Pathog. 2014; 10: e1004516 10.1371/journal.ppat.1004516 25474113 PMC4256457

[75] Veal EA, Day AM, Morgan BA. Hydrogen peroxide sensing and signaling. Mol Cell. 2007; 26: 1–14 17434122 10.1016/j.molcel.2007.03.016

[76] D'Autreaux B, Toledano MB. ROS as signalling molecules: mechanisms that generate specificity in ROS homeostasis. Nat Rev Mol Cell Biol. 2007; 8: 813–824 17848967 10.1038/nrm2256

[77] Sobotta MC, Liou W, Stocker S, Talwar D, Oehler M, Ruppert T, et al Peroxiredoxin-2 and STAT3 form a redox relay for H_2_O_2_ signaling. Nat Chem Biol. 2015; 11: 64–70 10.1038/nchembio.1695 25402766

[78] Guevara AG, Guilvard E, Borges MM, Cordeiro da SA, Ouaissi A. N-Acetylcysteine and glutathione modulate the behaviour of *Trypanosoma cruzi* experimental infection. Immunol Lett. 2000; 71: 79–83 10714433 10.1016/s0165-2478(99)00164-9

[79] Burleigh BA, Andrews NW. The mechanisms of *Trypanosoma cruzi* invasion of mammalian cells. Annu Rev Microbiol. 1995; 49: 175–200 8561458 10.1146/annurev.mi.49.100195.001135

[80] de SW, de Carvalho TM, Barrias ES. Review on *Trypanosoma cruzi*: Host Cell Interaction. Int J Cell Biol. 2010; 2010 10.1155/2010/295394 PMC2926652 20811486

[81] Dhiman M, Garg NJ. NADPH oxidase inhibition ameliorates *Trypanosoma cruzi*-induced myocarditis during Chagas disease. J Pathol. 2011; 225: 583–596 10.1002/path.2975 21952987 PMC4378678

[82] Castor LR, Locatelli KA, Ximenes VF. Pro-oxidant activity of apocynin radical. Free Radic Biol Med. 2010; 48: 1636–1643 10.1016/j.freeradbiomed.2010.03.010 20304045

