# Supplementary figures and images for: Trypanosoma cruzi Needs a Signal Provided by Reactive Oxygen Species to Infect Macrophages
Source: PLoS Negl Trop Dis. 2016 Apr 1;10(4):e0004555. doi: 10.1371/journal.pntd.0004555 (PMC4818108; doi:10.1371/journal.pntd.0004555)

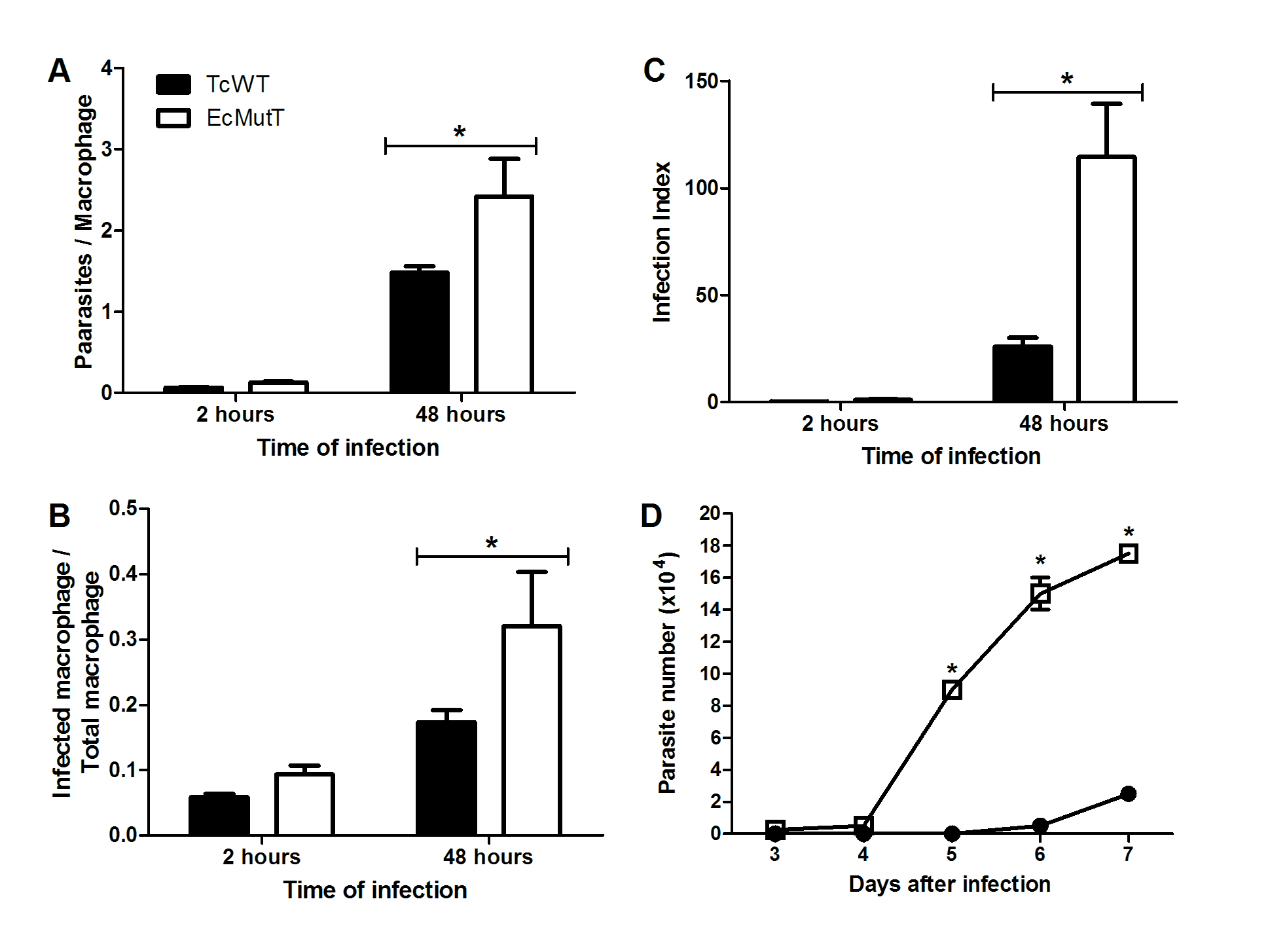

Supplement: S1 Fig — Inflammatory macrophages obtained from peritoneal cavity of C57BL/6 WT mice were infected with wild type and modified parasites. The cells were washed to remove extracellular parasites and either fixed or re-incubated with medium for different times. (A) Number of parasites per macrophage. (B) Number of infected macrophage per total macrophage. (C) Infection index for each parasite population. (D) Number of parasites released into the macrophage culture supernatant between the third and seventh day after infection. Data shown are representative of a second of three independent experiments performed in triplicate (cells were pooled from three mice for each replicate). All data are presented as the means ± standard deviation. * indicates significant differences between marked bars or between points, p<0.05, two-way ANOVA test with Bonferroni post-test. (TIF) [file pntd.0004555.s001.tif]

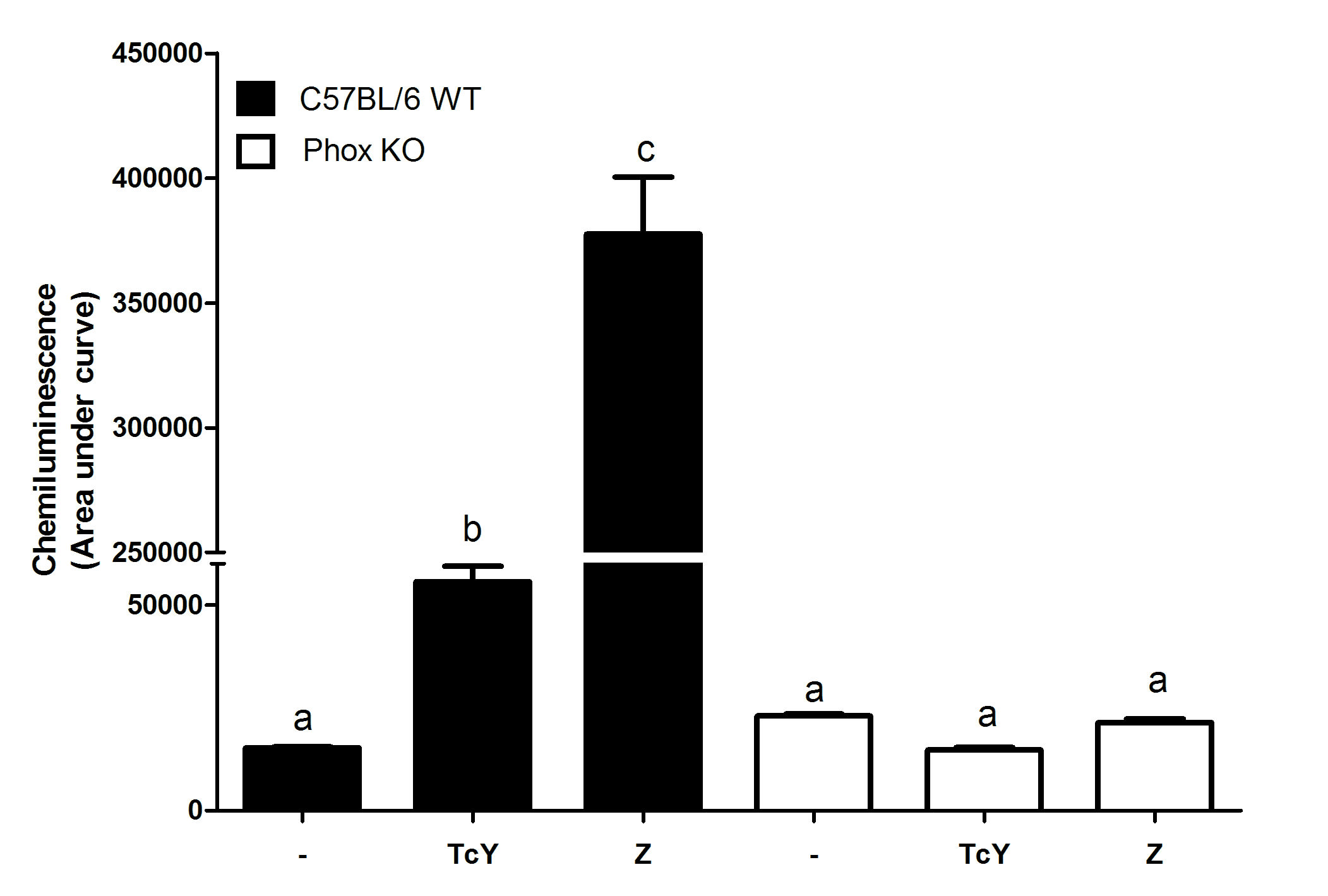

Supplement: S4 Fig — Thioglycolate-elicited macrophages were harvested from the peritoneal cavity of C57BL/6 WT and Phox KO mice 4 days after stimulation. Reactive oxygen species production by macrophages was detected by luminol. Chemiluminescence was continuously measured immediately after T. cruzi (TcY) or zymosan (Z, 1x10^7^U/well) addition to the macrophage monolayer, and the area under the obtained curves was calculated. The graphs are representative of three independent experiments performed in triplicate (cells were pooled from three mice for each replicate). Bars marked by different letters are statistically different (p<0.05, one-way ANOVA test with Bonferroni post-test). (TIF) [file pntd.0004555.s004.tif]
